# Sea level fingerprinting of the Bering Strait flooding history detects the source of the Younger Dryas climate event

**DOI:** 10.1126/sciadv.aay2935

**Published:** 2020-02-26

**Authors:** T. Pico, J. X. Mitrovica, A. C. Mix

**Affiliations:** 1 Department of Earth and Planetary Sciences, Harvard University, Cambridge, MA 02138, USA.; 2 Division of Geological and Planetary Sciences, California Institute of Technology, Pasadena, CA 91125, USA.; 3 College of Earth, Ocean, and Atmospheric Sciences, Oregon State University, Corvallis, OR 97331, USA.

## Abstract

During the Last Glacial Maximum, expansive continental ice sheets lowered globally averaged sea level ~130 m, exposing a land bridge at the Bering Strait. During the subsequent deglaciation, sea level rose rapidly and ultimately flooded the Bering Strait, linking the Arctic and Pacific Oceans. Observational records of the Bering Strait flooding have suggested two apparently contradictory scenarios for the timing of the reconnection. We reconcile these enigmatic datasets using gravitationally self-consistent sea-level simulations that vary the timing and geometry of ice retreat between the Laurentide and Cordilleran Ice Sheets to the southwest of the Bering Strait to fit observations of a two-phased flooding history. Assuming the datasets are robust, we demonstrate that their reconciliation requires a substantial melting of the Cordilleran and western Laurentide Ice Sheet from 13,000 to 11,500 years ago. This timing provides a freshwater source for the widely debated Younger Dryas cold episode (12,900 to 11,700 years ago).

## Teaser

Two-phased flooding of Bering Strait is reconciled by melting North American ice saddle during Younger Dryas.

## INTRODUCTION

The timing of the last separation between the major North American ice sheets is central to paleoclimate studies and to arguments that an ice-free corridor served as a path for early human migration. However, efforts to constrain the history of retreat between the Laurentide and Cordilleran ice sheets have been limited by sparse geologic observations (*1*). Glacial isostatic adjustment due to the disintegration of this ice saddle and removal of its mass load on the continent would induce a local sea level fall and exert an important influence on sea level in the Bering Strait (*2*), ~2000 km to the west of the saddle region. Submergence of the Bering Strait modulates ocean circulation as it provides a return pathway for relatively low-salinity Pacific waters to the North Atlantic via the Arctic Ocean (*3*). The timing of this initial connection is thus a key issue in reconstructing past ice extent, as well as ocean and climate variability. However, this timing remains contentious; observations in sediment cores support an ~11.5-ka-old submergence of the Bering Strait (*4*–*6*), whereas other lines of evidence based on marine species dispersal suggest a connection 2 ka earlier at 13.3 ka ago (*7*–*9*).

Here, we reconcile these apparently contradictory datasets related to the inundation of the Bering Strait using a gravitationally self-consistent relative sea level simulation based on an ice history that sources a substantial contribution of meltwater from the western Laurentide Ice Sheet (LIS) and the Cordilleran Ice Sheet (CIS), in the region west of 110°W, from 13 to 11.5 ka ago. Our proposed ice-melting scenario is consistent with the hypothesis that discharge of freshwater into the Arctic slowed thermohaline circulation and triggered the Younger Dryas cold episode (*10*), an abrupt climate event associated with a reduced Atlantic Meridional Overturning Circulation, from 12.9 to 11.7 ka ago. Our sea level reconstructions predict an evolution of regional shorelines that provides a key boundary condition for studies of human migration into the Americas.

### Observations of Bering Strait resubmergence

Current understanding of the timing of the Bering Shelf resubmergence during the last deglaciation is based on dated sediment cores that record local subaerial or marine environments, as well as geochemical shifts implying a change in ocean connectivity (Table 1; see Methods and sections S8.1 to S8.6 for radiocarbon calibration). The present sill depth at the Bering Strait is 53 m (*11*). If we assume minimal vertical displacement due to longer-term tectonic, erosion, and sedimentation effects (see section S1), the local sea level must have reached this elevation for the Arctic and Pacific oceans to be connected.

**Table 1:** Observational data and calibrated radiocarbon ages.

**Reference**	**Site** **name**	**Latitude**	**Longitude**	**Material**	**Marker** **depth (m)**	**Raw ^14^C** **age** **(years)**	**Raw** **age** **error**	**Δ*R***	**Δ*R*** **error**	**Calendar** **year** **(before** **present)**	**1σ−** **years**	**1σ+** **years**	**Calibration** **curve**
Jakobsson *et al.* (*4*)	4PC-1	72.8	175.7	Mollusc	124.07	10,200	30	250	200	10,900	230	510	Marine13
Keigwin *et al.* (*5*)	JPC02	67.4	165.6	*Elphidium* *excavatum*	53.455	10,900	140	250	200	11,950	350	490	Marine13
Hill and Driscoll (*6*)	VBC03*	70.7	165.4	Marine bivalve (Portlandia)	60.42	11,500	765	250	200	12,590	1120	910	Marine13
	JPC10	70.8	165.5	Marine bivalve	59.2	10,200	55	250	200	10,910	240	250	Marine13
Elias *et al.* (*12*)	85-69	70	165.7	Screened peat	44.95	11,000	60	0	0	12,870	90	80	Intcal13
Dyke and Savelle (*8*)				Bowhead whale bone		10,210	70	320	50	10,850	140	130	Marine13
Dyke *et al.* (*9*)				*Macoma* *balthica*		11,400	100	320	50	12,600	90	110	Marine13
England and Furze (*7*)				*Cyrtodaria* *kurriana*		12,380	110	320	50	13,520	140	130	Marine13
England and Furze (*7*)				*C. kurriana*		12,170	25	320	50	13,320	60	60	Marine13
England and Furze (*7*)				*C. kurriana*		11,800	70	320	50	12,960	110	121	Marine13

*Portlandia arctica raw ^14^C age reflects correction (800 ± 700 years subtracted from published raw ^14^C age).

**Fig. 1:**
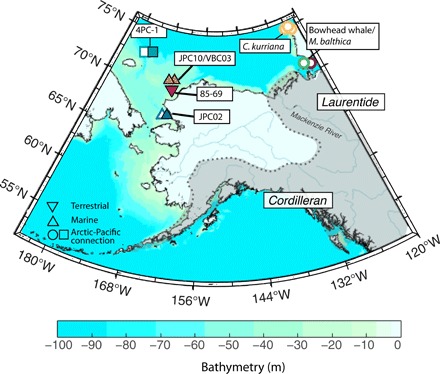
Bathymetry in the Bering Shelf region from ARDEM (*11*). Locations of observations constraining the timing of the Bering Strait resubmergence are shown by circles, triangles, and squares, which correspond to markers in Figs. 2 and 5. Pacific species found on uplifted beach terraces were collected on Victoria and Banks Island, Canada (*7*–*9*). Dotted gray line and shaded region show schematic of Last Glacial Maximum ice margin [Dyke (*1*)].

Elias *et al.* (*12*) dated terrestrial peat at a current water depth of 45.0 m on the Beringian continental shelf to 12.9 + 0.08/−0.09 (1σ) cal ka at site 85-69 (Figs. 1, red triangle, and 2), suggesting inundation of the Bering Strait later than this time. A transgressive flooding surface in core JPC10 from the Chukchi Shelf was analyzed by Hill and Driscoll (*6*), and an age of 10.9 + 0.25/−0.24 (1σ) cal ka was measured 1 m above the flooding surface at −59.2 m, although it was not possible to directly date the surface (Figs. 1, orange triangle, and 2). In nearby core VBC03, a shell contained within a mud clast was dated to 12.6 + 0.9/−1.1 (1σ) cal ka ago at 60.4-m depth (*6*), suggesting that relative sea level in the vicinity was higher than this depth by this time (Figs. 1, orange triangle, and 2). Keigwin *et al.* (*5*) inferred a rapid change from estuarine to open marine conditions at 12.0 + 0.5/−0.4 (1σ) cal ka ago from an increase in δ^18^O values above a flooding surface in sediment core JPC02 in Hope Valley, just north of Bering Strait, at 53.5-m depth (Figs. 1, overlapping open and filled blue triangles, and 2). Jakobsson *et al.* (*4*) inferred an inundation at ~11 ka ago based on a sharp increase in measured δ^13^C_org_ and biosilica content [with low values from at least ﻿~12.6 cal ka ago (*13*)], proxies for increased marine organic carbon input and Pacific influence, respectively, which was dated to 10.9 + 0.5/−0.2 (1σ) cal ka ago in core 4PC-1 from 120 m deep in Herald Canyon (Figs. 1, overlapping open and filled teal squares, and 2). Observations by Keigwin *et al.* (*5*) and Jakobsson *et al.* (*4*) point to a flooding event of age 11 to 11.5 ka and suggest that before this time, the Bering Strait sill separated the Pacific from the Arctic Ocean.

**Fig. 2:**
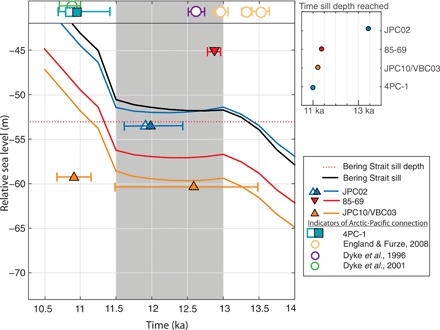
Relative sea level predictions for sites in the Bering Strait region compared with observations on the timing of Bering Strait resubmergence. Predictions at the Bering Strait sill are shown by the black line. Present-day sill depth is shown by horizontal dotted red line at −53 m. Upward-pointing triangles denote dates on marine deposits, implying relative sea level higher than the plotted elevation. Downward-pointing triangles denote terrestrial deposits, implying relative sea level lower than the plotted elevation. Open circles are observations signaling an open connection at the Bering Strait (e.g., Pacific species found in the Arctic), which means that Bering Strait had flooded sufficiently for tidal flows to allow species dispersal in a planktonic larval (veliger) stage, whereas squares are observations signaling significant flows through the Bering Strait. Error bars represent 1σ uncertainties (see the Supplementary Materials). Overlapping filled and open squares or triangles represent geochemical evidence of a transition from a closed to a significantly open connection at the Bering Strait between the Arctic and Pacific Oceans with net northward water flows. The gray rectangle highlights the interval from 13 to 11.5 ka ago. The blue, red, and orange lines show relative sea level histories at the corresponding site of observation (Fig. 1) using ice history GI-31.

The initial appearance of Pacific species in the Arctic provides an independent constraint on the timing of the Bering Strait inundation. Dyke and Savelle (*8*) and Dyke *et al.* (*9*) found species endemic to the Pacific on raised beach terraces in the Canadian Arctic, specifically bowhead whale bones and the mollusk *Macoma (Limecola) balthica*, and dated these specimens, respectively, to 10.9 ± 0.1 and 12.6 + 0.1/−0.9 (1σ) cal ka ago (Fig. 2, open green and purple circles). More recently, England and Furze (*7*) dated three samples of *Cyrtodari kurriana*, a mollusk species likely migrating from the North Pacific, on Banks Island, Canada to 13.0 ± 0.1, 13.3 ± 0.6, and 13.5 ± 0.1 (1σ) cal ka ago (Fig. 2, yellow circles), demonstrating that a Pacific-Arctic connection existed at least 2 ka earlier than suggested by sediment cores that capture a transition indicating the submergence of the Bering Strait.

These two lines of evidence, sediment core records and species dispersal, point to initial times of Bering Strait inundation of ~11.5 and ~13.3 ka ago, respectively, separated by nearly 2 ka. While the absolute timing in both datasets depends on the reservoir ages for radiocarbon dates (see section S8), the relative timing does not. These apparently contradictory datasets suggest the possibility of a double opening of the Bering Strait, implying a local sea level fall or stillstand from 13 to 11.5 ka ago, an interval when globally averaged sea level rose at a pace in excess of 10 m/ka (*14*). In the next section, we explore whether ice-melting scenarios exist that produce a geographically variable sea level history in the Bering Strait region consistent with this entire set of observations.

## RESULTS

### Refining ice melt histories and reconstructing local sea level

#### Initial glacial isostatic adjustment modeling

**Fig. 3:**
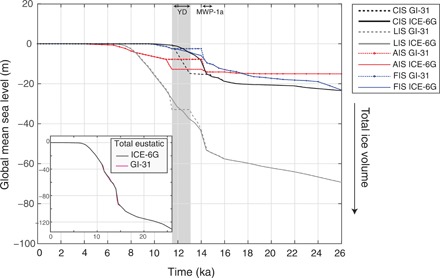
Eustatic contributions of each ice sheet. Cordilleran and western Laurentide Ice Sheet (CIS) in black, Laurentide Ice Sheet (east of 110°W; LIS) in gray, Fennoscandian Ice Sheet (FIS) in blue, and Antarctic Ice Sheet (AIS) in red, for both ICE-6G (solid) and an alternate ice model, GI-31 (dashed). The shaded gray rectangle highlights the interval of 13 to 11.5 ka. The Younger Dryas (YD; 12.9 to 11.7 ka) and Meltwater Pulse-1a (MWP-1a; 14.5 to 14 ka ago) are labeled. The inset compares total global eustatic histories for ICE-6G (gray) and GI-31 (pink), which differ by less than 2 m from 13 to 11.5 ka ago.

The melting of ice sheets across the last deglaciation drove a complex spatiotemporal pattern of sea level change due to deformational, gravitational, and rotational effects of glacial isostatic adjustment. The following simulations are based on the sea level theory and pseudo-spectral algorithm described by Kendall *et al.* (*15*). These predictions require models for Earth’s viscoelastic structure and the history of ice cover. We begin by adopting the global ice history ICE-6G (*16*) (Fig. 3, black line, inset) coupled with an Earth model characterized by a lithospheric thickness of 48 km and upper and lower mantle viscosities of 5 × 10^20^ and 5 × 10^21^ Pas, respectively (see Methods). Using the standard ice model ICE-6G, relative sea level at the Bering Strait is predicted to reach present-day sill depth at 11.5 ka, consistent with observations in sediment cores but inconsistent with evidence of an earlier connection at 13.3 ka (fig. S1, black dashed line).

#### Geological evidence and climate-ice and sea level modeling of LIS/CIS retreat

**Fig. 4:**
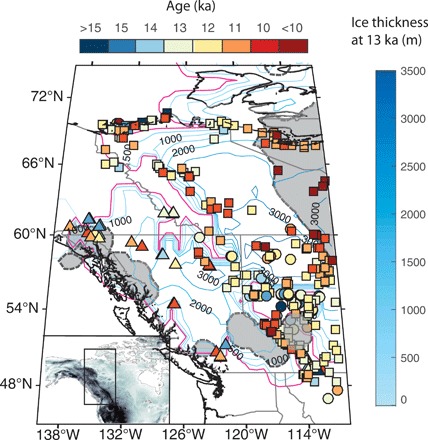
Ice melting scenario from 13 to 11.5 ka ago. Contours at 500-m intervals represent ice thickness at 13 ka ago in the GI-31 ice history (right-hand colorbar). Squares represent calibrated radiocarbon ages on organic material postdating ice loss, circles represent luminescence ages on aeolian dune deposits, and triangles represent cosmogenic ages on moraines, including cirque and valley moraines (see fig. S8). The GI-31 ice distribution from 15 to 13 ka ago is consistent with the median ages reported in this dataset (*n* = 818), constraining the deglaciation chronology of this region. For any dates that round to 13 ka ago, we ensure that these sites become ice free in the time step from 13 to 12.75 ka ago. Interior colors represent ages rounded to nearest integer (top colorbar). Dashed gray lines enclosing the areas filled with gray shading represent the limits of ice extent at 11.5 ka ago. Inset shows the map area in black rectangle. The magenta line shows the zero thickness contour at 13 ka ago.

Sea level in the vicinity of the Bering Strait is sensitive to the melting history of the CIS and the western LIS. Radiocarbon (Fig. 4, squares), luminescence (Fig. 4, circles), and cosmogenic dating (Fig. 4, triangles) have established minimum ages of ice retreat in this region (*17*–*20*). While there is evidence for ice-free conditions within the former saddle region by 15 ka (*19*), details of the timing, rate, and geometry of the saddle disintegration require refinement to understand the history of global mean sea level (GMSL) sourced from the eastern CIS and western LIS over the deglaciation. We use a compilation of 818 ages constraining the deglaciation of this region in our construction of ice-melt scenarios that are consistent with the inundation record at the Bering Strait (*17*–*20*) (Fig. 4; see section S7 for detailed discussion of ages).

Pulses of meltwater recorded in marine sediment cores provide additional constraints on the timing and location of ice ablation in the Cordilleran region. Davies *et al.* (*21*) observed similar early melting, with the retreat of the western CIS from its marine termination near ~14.8 cal ka and an increased freshwater influence in sediment cores in the Gulf of Alaska from 14.7 to 13.8 cal ka ago. In the Siberian Arctic, Spielhagen *et al.* (*22*) observed a freshwater peak in cores near 13 cal ka. Freshening in the Arctic near the Mackenzie delta, a direct outflow path from the former saddle region, is similarly observed in foraminiferal δ^18^O data from 12.9 to 12.2 cal ka ago (*23*) and from 12.7 to 11.6 cal ka ago (*24*, *25*) (see section S6 for updated ^14^C reservoir ages). Furthermore, regional seismic data and sedimentation patterns suggest a flood of meltwater at this time (*23*, *26*, *27*).

An ice sheet modeling study (*28*) predicted a rapid collapse of this ice sheet sector equivalent to a rise of 14.2-m GMSL over 2 ka, with a peak of ~10.5 m in a 0.5-ka period. Although this main pulse of melting occurred at 11.6 ka ago in model time, it was assumed to have been coincident with Meltwater Pulse-1a (MWP-1a; 14.5 ka ago) (*28*). We evaluate this assumption in light of sea level observations on the Bering Shelf.

Melting of the CIS-LIS saddle results in a local sea level rise in the Bering Strait region significantly smaller than the global average, as ice sheet melting produces a near-field component of sea level fall due to a loss of gravitational attraction between the ocean and ice mass and crustal uplift due to ice unloading (*2*). Thus, a substantial melting of the CIS/western LIS extending from 13 to 11.5 ka ago could have been sufficient to produce a sea level fall or stillstand over this period. We hypothesize that such an event would reconcile observations of a possible double opening of the Bering Strait and would be consistent with other glaciological, geomorphological, and sea level evidence during this time interval.

#### Modification to ICE-6G: Fitting observations in the Bering Strait

We construct a hypothetical alternate ice model (GI-31) by modifying the deglaciation geometry of the ICE-6G model while requiring that the total ice volume (or GMSL) history of that model be preserved to satisfy far-field sea level constraints (see Fig. 3, inset, and section S9). We delay ice loss in the CIS/western LIS region within the zone west of 110°W, preserving the ICE-6G ice distribution at 15 ka ago in this specific region until 13 ka. The ice distribution from 15 to 13 ka ago is modified to be consistent with the median ages reported in a large dataset (*n* = 818) constraining the deglaciation chronology of this region. In particular, we require that regions with minimum ages older than 13 ka must be ice free by 13 ka ago in the GI-31 ice history (Fig. 4). A comparison with previously published ice models (*16*, *28*, *29*) and a map showing time steps of ice cover from 13 to 11.5 ka is included in figs. S2 and S3.

We source 14.3 m of GMSL rise from 13 to 11.5 ka ago from the CIS/western LIS region, which results in a total rate of GMSL rise from North American ice sheets (Fig. 3, black and gray lines) of 9.5 m/ka from 13 to 11.5 ka ago, slightly higher than the ICE-6G rate of 9.2 m/ka over the same interval. To maintain a total GMSL curve that matches the ICE-6G history, we revise this history such that melting of the Antarctic Ice Sheet (AIS) equivalent to a GMSL rise of 5.7 m from 13 to 11 ka ago and melting of the Fennoscandian Ice Sheet (FIS) equivalent to a GMSL rise of 5.3 m previously inferred to occur from 13 to 11.5 ka ago are assumed to occur earlier, during MWP-1a (~14.5 to 14.0 ka ago; Fig. 3, red and blue dashed lines; see section S4.4 for discussion on MWP-1a sources).

The ice model ICE-6G sources a large melt contribution across MWP-1a from a rapid ice margin retreat between the CIS and LIS equivalent to 6.9-m GMSL from 14.5 to 14 ka ago and a total of 10.6 GMSL from 15 to 13 ka ago at a mean rate of 6.1 m/ka (*16*). This ice model also sources 10.2-m GMSL from the eastern LIS across MWP-1a from 14.5 to 14 ka ago and a total of 17.4 m GMSL from 15 to 13 ka ago. In contrast, ice model GI-31 sources 0-m GMSL from the CIS/western LIS from 14.5 to 14 ka ago across MWP-1a and a total of 2.4-m GMSL from 15 to 13 ka ago. The ice model GI-31 sources 10.2-m GMSL from the eastern LIS across MWP-1a, from 14.5 to 14 ka ago, and a total of 21.8 m GMSL from 15 to 13 ka ago. The only source of meltwater from 13 to 11.5 ka ago in the GI-31 ice model is the CIS/western LIS (Fig. 3, black dashed line).

The relative sea level prediction adopting ice model GI-31 at the Bering Strait sill (65.5°N, 168.7°W) exceeds the present-day sill depth at ~13.3 ka with a value of −51.6 m at 13 ka ago and remains near this elevation until 11.5 ka ago (increasing to −50.3 m), after which relative sea level increases rapidly (Figs. 2, black line, and 5). This sea level stillstand occurs because of a combination of a local sea level fall due to gravitational and deformation effects added to the GMSL change produced by the input ice history (fig. S4).

**Fig. 5:**
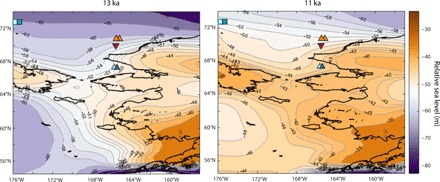
Map of relative sea level predictions at 13 and 11 ka ago. Sites with observations on resubmergence of the Bering Strait are shown by upward- and downward-pointing triangles, which denote marine or terrestrial deposits, respectively. Adjacent white boxes show dates associated with each observable. At 13 ka ago, the relative sea level at the Bering Strait sill is higher than the −53-m threshold; however, the relative sea level is lower at other sites on the Bering Shelf.

Next, we consider predictions of relative sea level at each core site plotted in Fig. 1. We note that relative sea level varies substantially (>10 m) throughout the Bering Shelf region because of the strong gradient in gravitational and deformation effects in the near field of the CIS/western LIS region (fig. S5). Therefore, relative sea level at each site exceeds the local present-day sill depth at different times (Fig. 2, inset), and this temporal variability is consistent with constraints imposed by the datasets summarized in Fig. 2. For example, at site 4PC-1, rapid sea level rise started at 11.5 to 11 ka ago as the Bering Strait sill deepened, consistent with the increase in Pacific water influence observed by Jakobsson *et al.* (*4*) (Fig. 2, teal squares). Moreover, the relative sea level at site VBC03/JPC10 is just above −60 m at 13.3 ka ago (Fig. 2, orange line), while the relative sea level at site 85-69 is below −45 m at 12.9 ka ago (Fig. 2, red line).

## DISCUSSION

Our calculations predict relative sea level to be higher near the sill than in the surrounding regions, explaining discrepancies in reported relative sea level elevations (see Figs. 2 and 5) that are not reconciled using the standard ICE-6G history (i.e., JPC10/VBC03; fig. S1). GI-31 predicts a first breach of the sill by 13 ka ago. Even a shallow inundation at the sill would allow dispersal of shallow-water mollusk species with a planktonic larval stage or bones from a deceased whale, and this can explain the early appearance of Pacific mollusk species in the Arctic. Total inundation of the Bering Strait does not occur in the simulation until 11.5 ka ago, explaining the later date for biosilica accumulation, which requires a sill depth great enough for substantial northward flows of Pacific water (Fig. 2).

### Sensitivity tests

We construct two additional ice histories that sample the range of ice-melting scenarios consistent with the age uncertainties associated with our data compilation. We adopt the 2σ error minimum (GI-34; “older scenario”; fig. S6A) and maximum (GI-30; “younger scenario”; fig. S6B) ages and use these as constraints to refine the ice-melting scenario from 13 to 11.5 ka ago. These reconstructions result in a smaller (12.6 GMSL) and larger (15.1 GMSL) ice volume loss in the CIS/western LIS region for the older and younger age scenarios, respectively, over the 13- to 11.5-ka ago interval, and result in relative sea level rises of 1.7 and 1.2 m, respectively, from 13 to 11.5 ka ago at the Bering Strait sill.

We test the robustness of these results to variations in the regional distribution and duration of ice melt by constructing a series of alternate ice models. Simulations varying the timing of subregions of melting within the period 13 to 11.5 ka ago suggest that a relative sea level stillstand at the Bering Strait required melting in the northwest region of CIS/western LIS over the entire duration of 13 to 11.5 ka ago (fig. S7B). We also performed calculations that explored the sensitivity of sea level predictions to the total mass flux from the CIS-LIS region, the AIS, FIS, and eastern LIS. These simulations indicate that the predicted relative sea level stillstand is robust to different relative contributions of these far-field ice sheets to MWP-1a (fig. S7B).

We assessed the sensitivity of the relative sea level predictions in the Bering Strait region to the adopted Earth model by varying the lithospheric thickness and upper and lower mantle viscosities. We found that while the Earth model modulates the amplitude of relative sea level, the total sea level rise from 13 to 11.5 ka ago is robust, varying by less than 0.7 m. This insensitivity is a consequence of the dominance of gravitational and elastic deformation effects (fig. S8).

There are a variety of datasets that can be brought to bear to refine the spatial geometry of ice loss local to the region of saddle melting, including the present-day elevation of proglacial lake shorelines and site-specific relative sea level histories (*29*). Since the ICE-6G ice history was not tuned to fit most of these records, the ice model GI-31 based on it would not be expected to fit these datasets. In the Supplementary Materials (see section S10), we adopt the model NAICE (*29*) that was, in contrast, tuned to fit these datasets and demonstrate that introducing the delay in saddle melting necessary to reconcile the Bering Strait sea level record does not introduce a misfit to the lake and relative sea level data in the vicinity of the ice loss (figs. S14 to S17).

## CONCLUSION

We refine the timing and geometry of relative sea level change in the Bering Strait during the last deglaciation by constructing an ice history within the CIS and western LIS that is consistent with available land dates. Our ice sheet reconstructions, which maintain fits to sea level records in the far-field (section S9), yield sea level predictions that reconcile disparate and previously enigmatic datasets recording the inundation history of the Bering Shelf. Our inferred ice-melting scenarios source substantial meltwater from the retreat between the CIS and LIS from 13 to 11.5 ka ago in the region west of 110°W, potentially initiated by marine retreat of the ice sheet (fig. S16). Part of the freshwater flux from this ice-mass loss (0.11 Sv over the period 13 to 11.5 ka ago) would have freshened the subpolar North Atlantic and may have been sufficient to suppress deepwater convection and thereby initiate Younger Dryas cooling (*30*–*32*). The end of the meltwater flux may have also had a role in terminating the anomalous Younger Dryas cooling and triggering the onset of early Holocene warmth (*33*).

## METHODS

### Radiocarbon date calibration

The calculation of Arctic ^14^C ages is somewhat uncertain north of the Bering Strait. Published reservoir ages range from ~450 to 900 years (i.e., Δ*R* from 50 to 500 years) (*4*, *34*, *35*). The value used in 4PC-1 is relatively low, Δ*R* = 50 ± 100 years (*4*) for the early inundation period based on assumed sea level; if a higher value is used, then the inferred date for sea level rise there could be as young as 10 ka. In contrast, the reservoir age used for the mollusks in the Canadian Archipelago is 1140 years [Δ*R* = 740 (*36*)]; if a younger reservoir age is applied there, then the first appearance of Pacific fauna in the Arctic could be as old as 14 ka. It is unlikely that plausible changes in reservoir age could collapse the spread of dates toward a single unified value.

Given these uncertainties, we recalibrated ^14^C raw ages to present data consistently throughout the text. We assigned a reservoir age anomaly (Δ*R*) of 320 ± 50 years to post-inundation Canadian Arctic ^14^C dates according to modern (prebomb) mollusk calibrations done by Coulthard *et al.* (*37*). On the Arctic side of the Bering Strait for dates between 12,000 and 10,000 years ^14^C, we assigned a Δ*R* = 250 ± 200 years. This reservoir age correction was selected as an intermediate between likely Δ*R* values before an Arctic-Pacific connection (Δ*R* = 60 ± 40) and likely values post-Bering Strait flooding (Δ*R* = 350 ± 120). See sections S6.1 to S6.6 for a detailed discussion of these reservoir ages. We used the Marine13 or Intcal13 for marine and terrestrial deposits, respectively (*38*). See Table 1 for the compilation of data.

### Glacial-isostatic adjustment modeling

Relative sea level calculations are based on the sea level theory and pseudo-spectral algorithm described by Kendall *et al.* (*15*) with a spherical harmonic truncation at degree and order 256. These calculations include the impact of load-induced Earth rotation changes on sea level, evolving shorelines and the migration of grounded, marine-based ice. Given the sensitivity of relative sea level amplitudes to the selected Earth viscosity profile model, we chose a viscosity profile based on (i) fitting relative sea level records in the Bering Shelf region shown in Fig. 2 and (ii) regional seismic studies that support a thinner than average crustal thickness in this area (*39*). Specifically, we adopted an Earth model characterized by a lithospheric thickness of 48 km and an upper and lower mantle viscosity of 5 × 10^20^ and 5 × 10^21^ Pas, respectively, consistent with previous inferences of mantle viscosity profiles (*40*). We used this radially symmetric one-dimensional viscosity profile rather than VM5a, a model designed to pair with ICE-6G, for predicted relative sea level to breach the Bering Strait by 11.5 ka ago. When we paired ICE6G with VM5a, we predicted a relative sea level of −49.1 m at 11.5 ka ago. We assessed the sensitivity to these model parameters in the Supplementary Materials (fig. S8).

## Supplementary Material

http://advances.sciencemag.org/cgi/content/full/6/9/eaay2935/DC1

Download PDF

Data file S1

Sea level fingerprinting of the Bering Strait flooding history detects the source of the Younger Dryas climate event

## SUPPLEMENTARY MATERIALS

Supplementary material for this article is available at http://advances.sciencemag.org/cgi/content/full/6/9/eaay2935/DC1 Section S1. Uncertainty on elevation of Bering Strait sill at 13 to 11.5 ka ago Section S2. Local observations of flooding as sea level markers Section S3. Contributions to relative sea level: Gravitational versus deformational effects Section S4. Sensitivity to ice model Section S5. Meltwater flux volumes to Arctic Ocean Section S6. Meltwater pulses recorded in Arctic Section S7. Terrestrial geologic data constraining CIS and LIS retreat Section S8. Radiocarbon reservoir age corrections Section S9. Fitting relative sea level constraints in far field Section S10. Fitting glacial lake shoreline tilts and local relative sea level histories Fig. S1. Relative sea level predictions at each site of observation for ice model GI-31 and ICE-6G adopting the Earth model described in the main text. Fig. S2. Ice thickness at 13 ka ago for various ice histories. Fig. S3. Snapshots of ice thickness from 13 to 11.5 ka ago for ice history GI-31 (ice history adopted in the main text). Fig. S4. Decomposition of total relative sea level at the Bering Strait sill into components associated with the direct gravitational effect of the surface load and crustal deformation, including the local gravitational effect of this deformation. Fig. S5. Map of the difference in relative sea level predictions at 13 ka BP predicted using the GI-31 and ICE-6G ice histories and the Earth model described in the main text. Fig. S6. Ice melting scenario from 13-11.5 ka ago for GI-34 and GI-30. Fig. S7. Relative sea level predictions based on a suite of ice models, testing the sensitivity of the predictions to changes in the regional distribution and duration of ice melt. Fig. S8. Earth model sensitivity. Fig. S9. Oxygen isotope record from planktonic foraminifera from Mackenzie delta. Fig. S10. The location of cirque and valley glacier moraines in the Menounos *et al.* (*18*) study is shown on Fig. 4. Fig. S11. Relative sea level predictions for sites in the Bering Strait region compared with observations using radiocarbon dates calibrated with additional uncertainty. Fig. S12. Relative sea level predictions using ice history GI-31, GI-30, and GI-34. Fig. S13. Comparison of measured and predicted tilt using ice history NAICE-D and NAICE. Fig. S14. Paleotopography compared to observed shoreline elevations. Fig. S15. Misfit between the observed and predicted paleotopography for each glacial Lake Agassiz shoreline. Fig. S16. Relative sea-level predictions compared with relative sea-level markers in the Arctic older than 11 ka ago. Fig. S17. Possible marine retreat of ice sheet. Table S1. Compilation of ice models used in this study. Table S2. Calibrated radiocarbon ages using a larger uncertainty on reservoir ages than in main text (Δ*R* = 300 ± 200 years). Data file S1. Compilation of ages constraining timing of ice retreat in CIS/Western LIS.
