# Supplementary material for: Sea level fingerprinting of the Bering Strait flooding history detects the source of the Younger Dryas climate event
Source: Sci Adv. 2020 Feb 26;6(9):eaay2935. doi: 10.1126/sciadv.aay2935 (PMC7043918; doi:10.1126/sciadv.aay2935)
Supplement: http://advances.sciencemag.org/cgi/content/full/6/9/eaay2935/DC1 [file supp_6_9_eaay2935__index.html]

Science Advances | Science AdvancesAAASSearchScience AdvancesMenu

## Supplementary Materials

**The PDF file includes:**

- Section S1. Uncertainty on elevation of Bering Strait sill at 13 to 11.5 ka ago
- Section S2. Local observations of flooding as sea level markers
- Section S3. Contributions to relative sea level: Gravitational versus deformational effects
- Section S4. Sensitivity to ice model
- Section S5. Meltwater flux volumes to Arctic Ocean
- Section S6. Meltwater pulses recorded in Arctic
- Section S7. Terrestrial geologic data constraining CIS and LIS retreat
- Section S8. Radiocarbon reservoir age corrections
- Section S9. Fitting relative sea level constraints in far field
- Section S10. Fitting glacial lake shoreline tilts and local relative sea level histories
- Fig. S1. Relative sea level predictions at each site of observation for ice model GI-31 and ICE-6G adopting the Earth model described in the main text.
- Fig. S2. Ice thickness at 13 ka ago for various ice histories.
- Fig. S3. Snapshots of ice thickness from 13 to 11.5 ka ago for ice history GI-31 (ice history adopted in the main text).
- Fig. S4. Decomposition of total relative sea level at the Bering Strait sill into components associated with the direct gravitational effect of the surface load and crustal deformation, including the local gravitational effect of this deformation.
- Fig. S5. Map of the difference in relative sea level predictions at 13 ka BP predicted using the GI-31 and ICE-6G ice histories and the Earth model described in the main text.
- Fig. S6. Ice melting scenario from 13-11.5 ka ago for GI-34 and GI-30.
- Fig. S7. Relative sea level predictions based on a suite of ice models, testing the sensitivity of the predictions to changes in the regional distribution and duration of ice melt.
- Fig. S8. Earth model sensitivity.
- Fig. S9. Oxygen isotope record from planktonic foraminifera from Mackenzie delta.
- Fig. S10. The location of cirque and valley glacier moraines in the Menounos *et al.* (18) study is shown on Fig. 4.
- Fig. S11. Relative sea level predictions for sites in the Bering Strait region compared with observations using radiocarbon dates calibrated with additional uncertainty.
- Fig. S12. Relative sea level predictions using ice history GI-31, GI-30, and GI-34.
- Fig. S13. Comparison of measured and predicted tilt using ice history NAICE-D and NAICE.
- Fig. S14. Paleotopography compared to observed shoreline elevations.
- Fig. S15. Misfit between the observed and predicted paleotopography for each glacial Lake Agassiz shoreline.
- Fig. S16. Relative sea-level predictions compared with relative sea-level markers in the Arctic older than 11 ka ago.
- Fig. S17. Possible marine retreat of ice sheet.
- Table S1. Compilation of ice models used in this study.
- Table S2. Calibrated radiocarbon ages using a larger uncertainty on reservoir ages than in main text (Δ*R* = 300 ± 200 years).
- Legend for data file S1

Download PDF

**Other Supplementary Material for this manuscript includes the following:**

- Data file S1 (Microsoft Excel format). Compilation of ages constraining timing of ice retreat in CIS/Western LIS.

**Files in this Data Supplement:**

- Adobe PDF - aay2935\_SM.pdf
