# Supplementary material for: Sea level fingerprinting of the Bering Strait flooding history detects the source of the Younger Dryas climate event
Source: Sci Adv. 2020 Feb 26;6(9):eaay2935. doi: 10.1126/sciadv.aay2935 (PMC7043918; doi:10.1126/sciadv.aay2935)
Supplement: Download PDF [file aay2935_SM.pdf]

Science Advances
AAAS

advances.sciencemag.org/cgi/content/full/6/9/eaay2935/DC1

Supplementary Materials for

Sea level fingerprinting of the Bering Strait flooding history detects the source of the Younger Dryas climate event

T. Pico*, J. X. Mitrovica, A. C. Mix

*Corresponding author. Email: tpico@caltech.edu

Published 26 February 2020, *Sci. Adv.* **6**, eaay2935 (2020)
DOI: 10.1126/sciadv.aay2935

The PDF file includes:

Section S1. Uncertainty on elevation of Bering Strait sill at 13 to 11.5 ka ago
Section S2. Local observations of flooding as sea level markers
Section S3. Contributions to relative sea level: Gravitational versus deformational effects
Section S4. Sensitivity to ice model
Section S5. Meltwater flux volumes to Arctic Ocean
Section S6. Meltwater pulses recorded in Arctic
Section S7. Terrestrial geologic data constraining CIS and LIS retreat
Section S8. Radiocarbon reservoir age corrections
Section S9. Fitting relative sea level constraints in far field
Section S10. Fitting glacial lake shoreline tilts and local relative sea level histories
Fig. S1. Relative sea level predictions at each site of observation for ice model GI-31 and ICE-6G adopting the Earth model described in the main text.
Fig. S2. Ice thickness at 13 ka ago for various ice histories.
Fig. S3. Snapshots of ice thickness from 13 to 11.5 ka ago for ice history GI-31 (ice history adopted in the main text).
Fig. S4. Decomposition of total relative sea level at the Bering Strait sill into components associated with the direct gravitational effect of the surface load and crustal deformation, including the local gravitational effect of this deformation.
Fig. S5. Map of the difference in relative sea level predictions at 13 ka BP predicted using the GI-31 and ICE-6G ice histories and the Earth model described in the main text.
Fig. S6. Ice melting scenario from 13-11.5 ka ago for GI-34 and GI-30.
Fig. S7. Relative sea level predictions based on a suite of ice models, testing the sensitivity of the predictions to changes in the regional distribution and duration of ice melt.
Fig. S8. Earth model sensitivity.
Fig. S9. Oxygen isotope record from planktonic foraminifera from Mackenzie delta.
Fig. S10. The location of cirque and valley glacier moraines in the Menounos *et al.* (*18*) study is shown on Fig. 4.

Fig. S11. Relative sea level predictions for sites in the Bering Strait region compared with observations using radiocarbon dates calibrated with additional uncertainty.
Fig. S12. Relative sea level predictions using ice history GI-31, GI-30, and GI-34.
Fig. S13. Comparison of measured and predicted tilt using ice history NAICE-D and NAICE.
Fig. S14. Paleotopography compared to observed shoreline elevations.
Fig. S15. Misfit between the observed and predicted paleotopography for each glacial Lake Agassiz shoreline.
Fig. S16. Relative sea-level predictions compared with relative sea-level markers in the Arctic older than 11 ka ago.
Fig. S17. Possible marine retreat of ice sheet.
Table S1. Compilation of ice models used in this study.
Table S2. Calibrated radiocarbon ages using a larger uncertainty on reservoir ages than in main text (ΔR = 300 $\pm$ 200 years).
Legend for data file S1

Other Supplementary Material for this manuscript includes the following:

(available at advances.sciencemag.org/cgi/content/full/6/9/eaay2935/DC1)

Data file S1 (Microsoft Excel format). Compilation of ages constraining timing of ice retreat in CIS/Western LIS.

SUPPLEMENTARY MATERIAL

Section S1. Uncertainty on elevation of Bering Strait sill at 13 to 11.5 ka ago

In this study we assume that in order for the Pacific and Arctic oceans to be connected relative sea level must reach -53 m, the present-day elevation at the Bering Strait sill. We further assume that minimal vertical displacements to the Bering Strait sill have occurred since 13 ka other than those associated with glacio-isostatic adjustment. However, a variety of processes besides glacio-isostatic adjustment can affect the paleoelevation of the sill, including tectonic activity, sedimentation and erosion, and changes in tidal or storm-surge regime. Estimates of tectonic activity suggest that less than 3-4 m of vertical displacement have occurred since 20 ka[41]. Given the absence of dated sediment cores directly on the sill, it is challenging to determine sedimentation rates at this site since 13 ka, nevertheless the core 85-69 cited in this study (Fig. 1) accumulated 1-4 m of sediment since flooding. At present-day the channel experiences relatively high current speeds, and therefore likely does not accumulate significant sedimentation[42]. An additional uncertainty is related to bathymetric resolution on the Bering Shelf. While present elevation at the Bering Strait sill is -53 m, the ARDEM bathymetry dataset suggests that there are partial sills to the north and south at -47 m elevation[40]. The effective sill depth for purposes of water transport may be somewhat shallower than the deepest passage through the sill. The effective sill depth for marine species dispersal may be similarly complex and species dependent.

In our simulations we show that varying the Earth model can modulate the absolute value of relative sea level. However, such changes do not affect whether a sea-level stillstand is predicted from 13-11.5 ka, which (given the constraint of global eustatic sea level rise at the time) is largely a response to the gravitational and elastic deformational effects of ice melt in the CIS/western LIS region. This is independent of the absolute value of relative sea level obtained with different Earth models.

Section S2. Local observations of flooding as sea level markers

In this study we synthesize published data including marine and terrestrial markers in addition to geochemical proxies for changes in water depth. While these datasets address the issue of the timing of initial Bering Strait flooding, our primary interest is reconstructing regional sea level rather than precisely dating the flooding surface at each site. While certain data sets we used were published including an interpolated or extrapolated age for the flooding surface (i.e. Hill & Driscoll[6]), we are primarily interested in these data as sea-level limiting indicators. Therefore, we use the calibrated dates of each sample and its observed elevation, rather interpolating or extrapolating an age for regional flooding based on individual locations. A key finding is that the apparent timing of local inundation is expected to vary between locations because of glacio-isostatic effects, so no single location is best suited to defining the timing at which sea level rose above the sill.

Section S3. Contributions to relative sea level: Gravitational versus deformational effects

Relative sea level at the Bering Strait is calculated as the sum of globally uniform sea-surface height variations and regional changes in relative sea level due to crustal deformation (including the impact of these changes on local sea-surface height) and the direct gravitational effects of the changing surface mass load. To assess the relative contribution of each process, we decomposed the relative sea level prediction based on ice models ICE-6G (dotted lines, fig. S4) and GI-31 (solid lines, fig. S4) into these two components: crustal deformation (red lines, fig. S4) and direct gravitational effects (blue lines, fig. S4). A large sea-level fall due to gravitational effects associated with the GI-31 model in the period 13-11.5 ka is a result of the contemporaneous loss of ice mass in the region at the border of the CIS and western LIS (henceforth the CIS-LIS zone). In the case of the ICE-6G simulation this sea level occurs over a longer period and earlier, during MWP-1a.

Figure S5 shows a map of the difference between relative sea level predictions for GI-31 and ICE-6G at 13 ka. relative sea level is higher in the region surrounding the CIS, western LIS and Bering Strait region for GI-31 from 15-13 ka because there is a larger ice mass in the CIS-LIS zone during this time compared with ICE-6G, and this exerts a gravitational pull on nearby oceans. The relative sea-level fall associated with the gravitational loss to this sector of the ice sheet from 13-11.5 ka occurs

rapidly in the GI-31 simulation. It is this component of relative sea level fall that results in the net sea-level stillstand predicted at the Bering Strait.

Section S4. Sensitivity to ice model

For the series of tests assessing sensitivity to the adopted ice model, we construct a simplified ice history GI-15, which has not been designed to as closely match geochronological constraints as GI-31, but rather consists of an ice-melting scenario from 13-11.5 ka in which the region west of 110°W in the CIS-LIS zone is retained from the ICE-6G history from 15 ka to 13 ka, and is then melted from 13-11.5 ka. Simulations using this ice history and adopting the Earth model in the main text results in a relative sea level rise of 1.2 m from 13-11.5 ka. We use this simplified ice melting scenario in order to assess sensitivity to the regional sources of melt, geometry of melt, and duration of melt.

4.1 Regional sources of meltwater from 13-11.5 ka

We assess the sensitivity of our results to regional distributions of ice melt. As an example, we shift the geographic limit of ice melt further east to 100° W (GI-19, purple line, fig. S7A), so as to broaden the zone of ice mass flux, and this results in an additional 1 m of sea-level rise across the 13-11.5 ka time window (2.2 m of sea-level rise compared with 1.2 m for GI-15).

We explore the sensitivity of sea-level in the Bering Strait to the location of ice melt by constructing alternative ice models where the southern or western region of the CIS-LIS zone melts from 13-12.25 ka, and the northern or eastern region melts in the remaining period from 12.25-11 ka. We find that during the period when the southern or eastern region is melting, sea level at the Bering Strait rises rapidly, resulting in a total sea-level change of 4.85 m (GI-23, blue line, fig. S7B) and 6.28 m (GI-26, light blue line, fig. S7B), respectively. Therefore, we conclude that a sea-level stillstand at the Bering Strait from 13-11.5 ka can be explained by melting from the northwestern section of the CIS-LIS zone over this entire interval, as this has the largest control on gravitational effects in the Bering Strait region.

We next consider how varying the timing of melting over the total CIS-LIS zone within the 13-11.5 ka period impacts relative sea level predictions. We modified the duration of melting to be longer (13-11 ka, GI-14, green line, fig. S7A) or shorter (12.5-11.5 ka, GI-18, blue line, fig. S7A), and this yielded an average rate of sea-level rise of 1.5 m/ky and 0.2 m/ky, respectively, compared with 0.8 m/ky for GI-15. By melting the majority of this ice in the first 0.5 ky we predict a sea-level fall of 0.7 m from 13-12.5 ka followed by a SL rise of 2.2 m until 11.5 ka (GI-12, orange line, fig. S7A).

4.2 Total GMSL required for sea-level stillstand

We construct additional ice models similar to GI-15, distinguished by smaller amounts of GMSL melt (50%, 75%, and 80%) from the CIS-LIS zone, where the remaining GMSL melt was sourced from the eastern sector of LIS (GI-24, purple, yellow, orange lines, fig. S7B), and find that these result in a sea level rise of 5.1 m, 3.0 m, and 2.6 m, respectively. If (for example) 3 m is an accepted value of sea-level rise over the 13-11.5 ka interval, we conclude that it is possible to source up to 25% of the total North American GMSL in the period 13-11.5 ka from the eastern LIS section. We construct two additional ice models that source 75% of the GMSL in CIS-LIS zone that melted in GI-15. In this case the remaining 25% of GMSL is sourced from the AIS (GI-28, magenta line, fig. S7B) or FIS (GI-27, crimson line, fig. S7B), to explore how the source of additional meltwater affects relative sea level in the Bering Strait. We find that latter results in a sea-level rise of 4.3 m, while the former predicts a sea-level rise of 3.7 m.

4.3 Saddle collapse vs. margin retreat

Ice modeling performed by Gregoire et al.[27] suggest a rapid saddle collapse of the CIS-LIS zone. The mechanism for this collapse involves rapid thinning of the larger region of the ice saddle, as surface melting lowers the ice surface causing an additional feedback of surface melting. Other authors have instead suggested an ice margin retreat[1], characterized by a separation between the two ice sheets, followed

by a retreat of the CIS and LIS ice margins. The model GI-15 (and GI-31, adopted in the main text) is based on the ice margin retreat associated with ICE-6G following the interpretation suggested by Dyke[1]. We construct an alternative model that adopts the saddle collapse (characterized by ice thinning, rather than margin retreat) using predictions by Gregoire et al[27]. We force total GMSL to be the same as GI-15 from the CIS-LIS region from 13-11.5 ka. This results in a total sea-level rise of 0.9 m (GI-25, green line, fig. S7B), which is smaller than the 1.2 m predicted by GI-15. We conclude that both these ice-melting hypotheses are consistent with observational data that suggests a sea-level stillstand or fall from 13-11.5 ka.

4.4 Sources of melt at MWP-1a

The meltwater sources for the rapid rise in sea level during MWP-1a are highly debated[13,43,44]. For purposes of a sensitivity test, we construct an additional ice model that tests whether our predictions of a sea-level stillstand at the Bering Strait are sensitive to where meltwater is sourced during MWP-1a. Similar to GI-15, this additional hypothetical ice model is based on ICE-6G. Instead of forcing the AIS and FIS to contribute to MWP1a, as in GI-15, we adopt the same AIS model as ICE-6G, and only modify the FIS component of the ICE-6G ice model. We require that ice melt from the FIS section of ICE-6G from 13-11.5 ka is sourced earlier at 14.5 ka. In this variation of GI-15, ice volumes in the CIS-LIS zone are preserved from 15 ka until 13 ka. In order to fit total global ice volumes, we require that the primary source for MWP-1a is the eastern sector of the LIS. Half of the ice volume lost from

14.5-13 ka in the eastern LIS sector in the ICE-6G model is forced to melt earlier from 14.5-14.25, so that this melt contributes to MWP-1a. This ice model, when paired with the Earth model adopted in the main text, yields relative sea level predictions in which sea level increases from -51.6 to -51.0 m from 13-11.5 ka (GI-22, red line, fig. S7B), with a total sea level rise of ~0.7 m (compared with 1.2 m for GI-15, solid black line, fig. S7).

This test indicates that a contribution of the AIS to MWP-1a, although possible[45], is not an essential factor in our construction of an ice history. Rather, our results rely solely on a small contribution from the CIS-LIS zone during MWP-1a (14.5-14.0 ka), and a large contribution during the Younger Dryas interval (13.0-11.5 ka).

4.5 Sensitivity to Earth model

We assessed the sensitivity of the relative sea level predictions in the Bering Strait region to variations in the adopted Earth model by varying the lithospheric thickness as well as the upper and lower mantle viscosities. We increased the lithospheric thickness to 96 km and found that relative sea level at 13 ka was shifted by 2.1 m (blue line, fig. S8). We next considered three Earth models in which the upper mantle viscosity was decreased to 3×10^{20} Pa s or the lower mantle viscosity was changed to either 7×10^{21} Pa s or 3×10^{21} Pa s. These three models perturbed relative sea level at 13 ka by 0.3 m, 1.9 m and -1.5 m, respectively (fig. S8).

Section S5. Meltwater flux volumes to Arctic Ocean

We can estimate the flux of freshwater created by melting ice in our various reconstructed ice histories. Ice model GI-31 (adopted in main text) sources 14.3 m GMSL from the CIS-LIS zone in 1.5 ky, resulting in a flux of 0.11 Sv (10^6 m^3/s). As a comparison, the duration of melt in ice model GI-18 is shortened to 0.5 ky, leading to a flux 0.33 Sv, melting the same volume in 2 ky results in a flux of 0.082 Sv. According to previously published estimates, this range of meltwater fluxes is sufficient to lead to a reduction in the strength of the Atlantic Meridional Overturning Circulation[10,31].

Section S6. Meltwater pulses recorded in Arctic

Our inference that ice loss in the CIS/western LIS region during the Younger Dryas interval predicts meltwater input directly the Arctic Ocean in the area of the modern Mackenzie River delta. We find support for this prediction in foraminiferal $\delta^{18}O$ data reported from sediment cores P189AR-P45 recovered from this region (70° 33.03'N, 141° 52.08'W, 405 m water depth west of the Mackenzie River[23]) and HLY1302-JPC 15 and JPC27 (JPC15: 71°06.222'N, 135°08.129'W; JPC27: 71°06.360'N, 135°09.640'W, 690 m water depth, east of the Mackenzie River[24]). We recalibrated ^{14}C measurements based on the reservoir age approach noted below in Supplementary Material 8, and calculated an age model that addresses analytical error, uncertainties in reservoir ages, and increased uncertainty between dated

levels in 10,000 BCHRON simulations[46]. Both sites document relatively low $\delta^{18}O$ of the planktonic foraminifera left-coiling *Neogloboquadrina pachyderma* (implying lower salinity likely forced by freshwater runoff) relative to a reconstruction of the global average marine $\delta^{18}O$ ice-volume component[47] between about 12,800 and 11,900 cal yr BP (fig. S9). The low- $\delta^{18}O$ (presumed meltwater) event is much stronger in the sites east of the Mackenzie River. The potential existence of older events is unconstrained in core P45, given the basal age of the core, but appear to be present (though less accurately dated) based on extrapolation of dates and a splice between two cores.

Section S7. Terrestrial geologic data constraining CIS and LIS retreat

The terrestrial deglaciation chronology of the late North American ice sheets is largely comprised of dates using ^{14}C, OSL, and cosmogenic methods (see Supplementary Excel Dataset). Radiocarbon dates on biological material provide a minimum age of ice retreat, as the location must have been ice-free to host vegetation. While the time required for a recently deglaciated area to harbor life is debated, some researchers have estimated as little as a 30 yr period[48]. OSL dates on aeolian dune deposits also involve a time lag, including the time required for a pro-glacial lake to retreat[18]. fig. S6 shows the 2σ uncertainty minimum and maximum on ages, and the corresponding ice melting scenarios for each (GI-34; GI-30).

Cosmogenic ages are used to record the time duration of exposure, since ice sheets (as well as snow cover) block cosmic flux radiation from penetrating the underlying rocks. These ages provide information on the last time that ice retreated from the region of observation. The type of moraine sampled contains important information on the style of glaciation. For instance, in Menounos et al.[17] the authors argue that dates on cirque and valley glacier moraines indicate that the style of ice cover had changed from extensive ice sheets to small alpine glaciers by 14 ka. These authors suggest that ages on cirque and valley glaciers indicate the CIS had fully deglaciated in this region, possibly thousands of years before alpine glaciation built cirque and valley glacier moraines.

We consider the sites presented in Menounos et al.[17] which are most relevant to our ice-melting scenario construction GI-31. In particular, the cirque moraine Site 9 has a mean age of ~14.3 ka (fig. S10; circle dot). If this age represents the timing of a total deglaciation of the Cordilleran Ice Sheet, then our ice history GI-31 can be ruled out. However, the actual set of dates obtained at this site show a large spread, ranging from 17.7 to 11.7 ka. The adjacent valley moraine Site 8 (fig. S10; triangle dot) includes a 40.84 ka age, suggesting this site may be prone to inheritance, opening the possibility that some of the older cirque dates at Site 9 include some inherited exposure.

A series of valley glacier sites (triangle dots; fig. S10) in the same vicinity, sites 10, 11, and 12, similarly show a large spread in ages, ranging from 10.24-14.81 ka,

10.16-19.45 ka, and 10.38-17.87 ka, respectively. However, because ^{10}Be was measured on small boulders, these samples may have been snow-covered and therefore should be corrected for this effect, suggesting that the snow and erosion corrected ages calculated in Menounos et al.[49], which are older than uncorrected dates, may be most accurate.

Section S8. Radiocarbon reservoir age corrections

Reservoir corrections to radiocarbon dates in the Bering Strait region are challenging and have been applied differently in various publications. Here we review available constraints and outline a framework for consistent application of reservoir age anomalies (ΔR) when correcting measured radiocarbon ages to calendar ages in this region.

8.1 "Portlandia" Effect

Many of the dates used to calibrate ^{14}C reservoir ages in the region are based on shallow-water mollusks. England et al.[50] note that infaunal deposit-feeding bivalves such as *Portlandia arctic*a yield ^{14}C ages as much as a few thousand years (uncertainty-weighted average recalculated here as 800 ± 700 yr) older than co-existing epifaunal filter feeders. They refer to this as the "Portlandia Effect", but this issue can also apply to deposit feeders such as *Macoma* sp., *Nuculan*a sp., and likely others. Suspension feeders such as *Astarte borealis*, *Musculus discors*, *Hiatella*

arctica, *Mytilus* sp., bivalves and *Balanus* barnacles appear to give more reliable dates. The *Portlandia* Effect is most severe in carbonate terranes where ^{14}C-free bedrock erodes to comprise a significant fraction of the ambient sediment and its subsequent diagenesis adds ^{14}C-free (aka "dead") carbon to the porewaters. Even in non-carbonate terranes the dates on infaunal taxa can be influenced by older organic matter and porewater effects. We have excluded infaunal taxa from our analysis to the extent possible, or make corrections as noted below.

8.2 Canadian Arctic reservoir age

Mollusk calibrations in the western Canadian Arctic[36] use corrections from the Intcal09 atmosphere[51], and exclude deposit feeders. They recommend a modern ΔR value of 335 ± 85 years. We recalculate these values based on Marine13[37] using the ΔR tool in CALIB-7.1[52], which calculates the ΔR value between a measured date and the Marine13 nominal ocean ^{14}C history. We only use samples collected prior to 1950, to avoid potential contamination with bomb radiocarbon, and we calculate an uncertainty-weighted average ΔR of 320 ±50 yr for the western Canadian Arctic.

8.3 Siberian Arctic reservoir age

On the Siberian Arctic margin, radiocarbon content of pre-bomb mollusks (excluding *Portlandia* sp.) in the Barents and Kara seas yield ΔR, recalculated here based on uncertainty-weighted averages, of -40 ± 40 yr [53]. In the Laptev Sea, a

similar study yields ΔR, recalculated here based on uncertainty-weighted averages as 60 ± 40 yr[34].

8.4 Bering Sea reservoir age

Radiocarbon reservoir effects are much larger in the Bering Sea, south of Bering Strait, because of the dominance of upwelled Pacific water, and limited gas exchange. Cook et al.[54] assumed ΔR values there of 300 yr based on published pre-bomb shell estimates ranging from 40-360 yr, but did not specify an uncertainty and stated that values may be higher in the past. Dumond and Griffin[55] reported apparent reservoir ages of marine shells relative to terrestrial carbon (charcoal, wood, or grass) of 460 ±35 yr (i.e., ΔR of about 50 ± 45 yr) and between marine mammal residue and terrestrial carbon of 735 ±20 yr (ΔR of about 325 ±30 yr). Although uncertain, these authors considered the larger value from the marine mammals to be more representative of the Bering Sea, and that the low values from shells may represent local effects.

Larger values from the Bering Sea are consistent with estimated Holocene values from the subpolar North Pacific in the Alaska Current (which flows into the Bering Sea), e.g., ΔR of 470 ± 80 yr[56] based on average benthic-planktonic age differences and modern deep-water ages, which are generally consistent with paleomagnetic correlations[57]. Regional correlations of deglacial paleoclimate records suggest a ΔR of 390±50 yr at the same site[58]. Tephrochronology constraints along the

southeastern Alaska margin during the deglacial interval yield a ΔR of 190 ±50 yr[59], while nearby comparisons of the age differences between terrestrial plant materials and marine bivalves yield a ΔR of 330±50 years[60]. The data from the Alaska margin and the Bering Sea together allow for variations in reservoir ages of a few hundred years, with ΔR values generally in the range of 350 ± 120 yr.

8.5 Reservoir ages adopted in this study

In this study we follow a strategy similar to Jakobsson et al.[4] in correcting marine radiocarbon dates for changing reservoir ages during the opening event, however we update calculations of these effects. We apply this strategy consistently to other published data. For radiocarbon dates on the Arctic side of Bering Strait, if clearly older than the inundation event or with ^{14}C dates > 12000 yr BP, we use the Laptev Sea ΔR of 60 ± 40 yr. For ^{14}C dates clearly younger than the inundation events with a substantially open Bering Strait or with ^{14}C dates < 10,000 yr BP we use the North-Pacific and Bering ΔR composite value of 350 ±120 yr. For times intermediate between a fully closed and open Bering Strait (the majority of the data included in Table 1 and Fig. 2), we are less certain about reservoir ages, and use an intermediate value of 250 ± 200 yr, acknowledging that this is not fully constrained.

For dating the initial occurrence of Pacific mollusk taxa in the Arctic we use the modern ΔR from the western Canadian archipelago of 320±50 years. For mollusks identified as infaunal deposit feeders (*Portlandia* sp., *Macoma* sp., or *Nuculana* sp.)

we subtract 800±700 yr from the measured radiocarbon date prior to applying a ΔR value. We calculate all calendar ages for marine materials based on the Marine13 database and Calib 7.1 software[52].

For calibrating calendar ages in terrestrial peats, we use the Intcal13 database and Calib 7.1 software, considering only wet-sieved peats that minimize contamination with older bulk carbon[11]. No reservoir age is applied to the peat samples. Note however, that different fractions of peat samples may yield different dates, either too young due to root penetration, or too old due to respired CO_2 from older deposits contributing to peat growth[61], implying that analytical uncertainties may underestimate geological uncertainty.

8.6 Calibrating ages with additional uncertainty

In addition to calibrating raw ^{14}C ages with the reservoir ages described above, we also calibrated ages using a reservoir age with a large uncertainty (ΔR = 300±200 yr). These calibrated ages are shown in table S2 and fig. S11.

Section S9. Fitting relative sea level constraints in far field

The reconstructed ice histories adopted in this study are characterized by GMSL histories that closely match the ICE-6G GMSL history. We predict relative sea level histories at Tahiti and Barbados for GI-31 and ICE-6G, adopting Earth model VM2 (Peltier & Fairbanks 2006) and find that differences between these predicted

relative sea level histories from 13-11.5 ka are less than 1.5 m. Therefore our modifications to the ICE-6G ice history in constructing GI-31 are well within the uncertainties associated with the well-established sea-level records at these sites (~5-10 m[13]).

Section S10. Fitting glacial lake shoreline tilts and local relative sea level histories

Glacial lake shorelines constitute an important geologic constraint on glacial isostatic adjustment, and recently published ice models have begun to incorporate such datasets in modeling of the glacial isostatic adjustment process[28,62]. The ice model NAICE[39], as an example, was constructed to yield predictions consistent with differential lake level changes and strandline tilts for glacial lakes throughout central and northwestern Canada. As a test, we construct a new ice model NAICE-D where we adopt a modified version of the NAICE ice model in which ice melting in the CIS- LIS region is delayed from 15 to 13 ka (as in GI-31, main text). We compared predictions based on the NAICE and NAICE-D ice histories, both coupled to an Earth model characterized by a 96 km thick elastic lithosphere, and upper and lower mantle viscosities of 4×10^{20} Pa s and 10^{22} Pa s, respectively (similar to Gowan et al.[28]).

We focused this test on data from Glacial Lake Agassiz because its shoreline features have been most reliably dated (Lepper et al., 2013, Yang and Teller, 2012). Since the Upper Campbell shoreline (~10.5 ka) contains the most extensive record of all the

Glacial Lake Agassiz shorelines, we compare the tilt of the shorelines predicted using the NAICE and NAICE-D models to observations (fig. S13; compare to Fig. 4 in Gowan et al.[28]). The NAICE model results are shown in green and the results based on the modified NAICE-D model are shown in blue. There are small differences in the predicted tilt, and both models perform well in fitting the measured tilt of the Upper Campbell strandline. Next, we can compare the predicted paleotopography at any relevant shoreline with the observed measured elevation. Figure S14 compares the paleotopography predicted using the two ice histories for the following Lake Agassiz shorelines: Upper Campbell (10.5 ka), Norcross (13.5 ka), and Herman (14 ka). To compare these predictions in more quantitative terms, we plot below the misfit between the predicted and observed elevations (fig. S15). Although these misfits show some variation between the predictions for the NAICE to NAICE-D models of ice history, these misfits are of the same magnitude.

Finally, we show relative sea level predictions using these two ice models and compare these predictions to observations of relative sea level in the Canadian Arctic dated to older than 11 ka (fig. S16). The results in figs. 15-17 demonstrate that introducing a delayed melting of ice from within the CIS-LIS region does not impact the fit to the local glacial lake and relative sea level data sets when one begins with a local ice history that fits these data.

SUPPLEMENTARY FIGURES

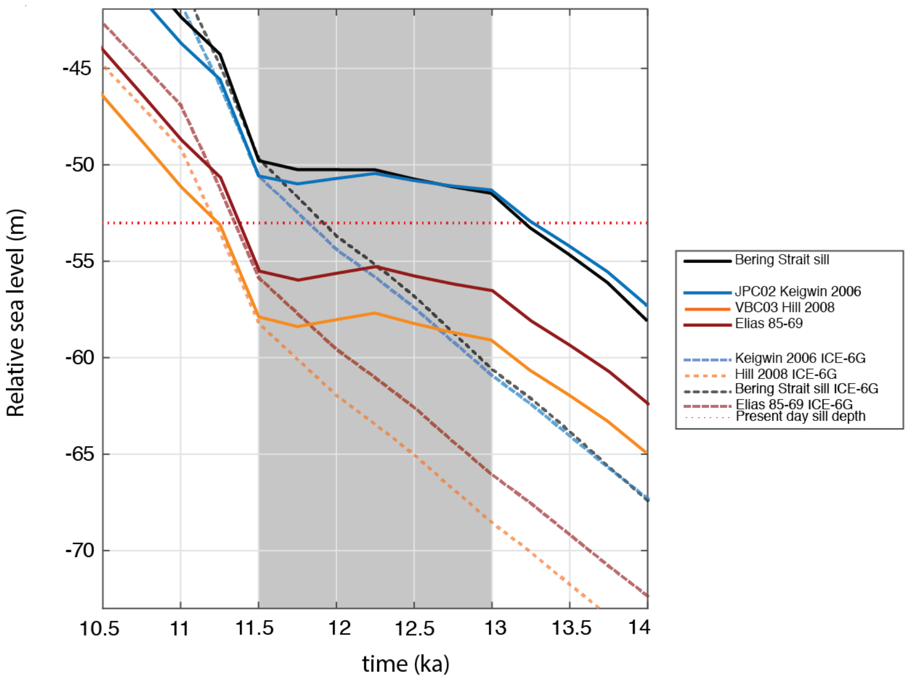

Fig. S1. Relative sea level predictions at each site of observation for ice model GI-31 (solid) and ICE-6G (dashed) adopting the Earth model described in the main text. The horizontal dotted red line represents present-day depth of the Bering Strait sill at -53 m.

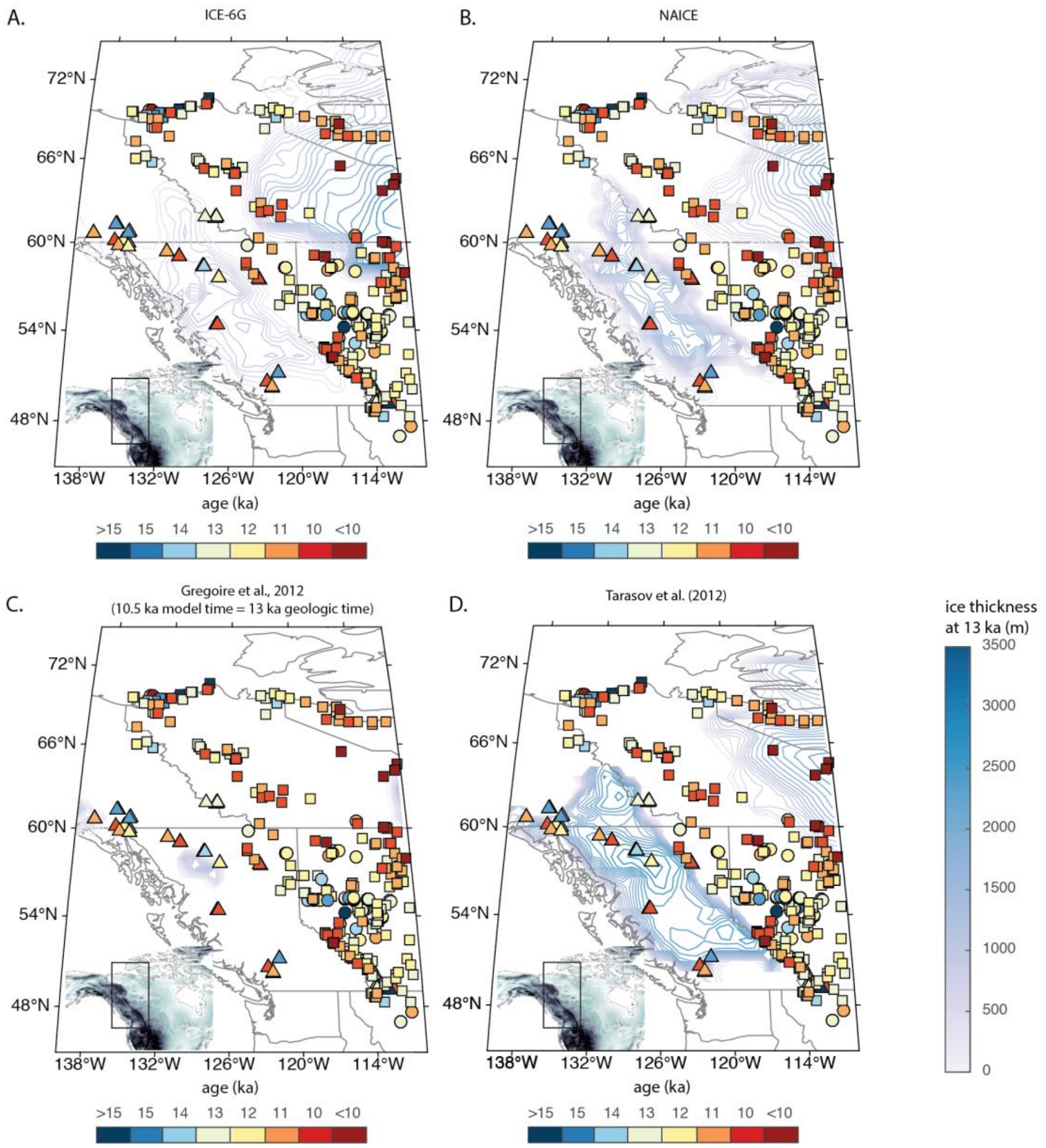

Fig. S2. **Ice thickness at 13 ka ago for various ice histories.** Ice thickness at 13 ka, analogous to Fig. 4 in the main text, except for the following ice histories: ICE-6G (**A**), NAICE (**B**), Gregoire et al., 2012 (**C**), and Tarasov et al., 2012(**D**).

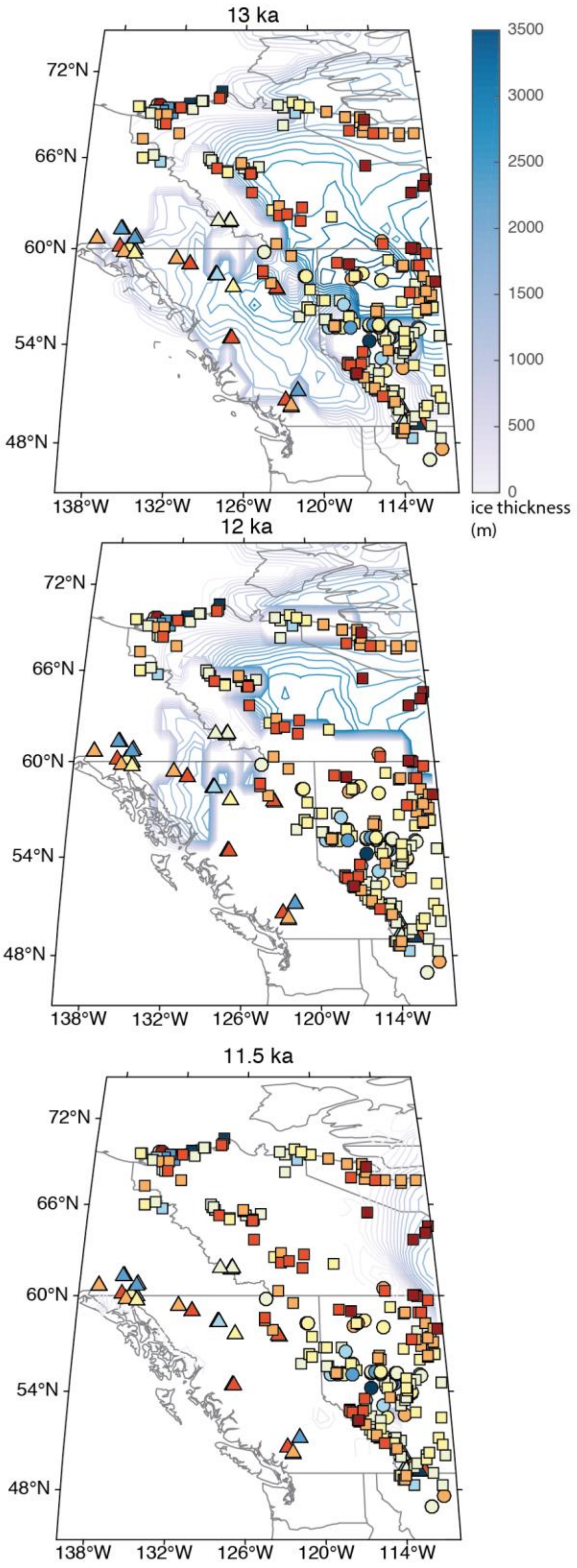

Fig. S3. Snapshots of ice thickness from 13 to 11.5 ka ago for ice history GI-31 (ice history adopted in the main text).

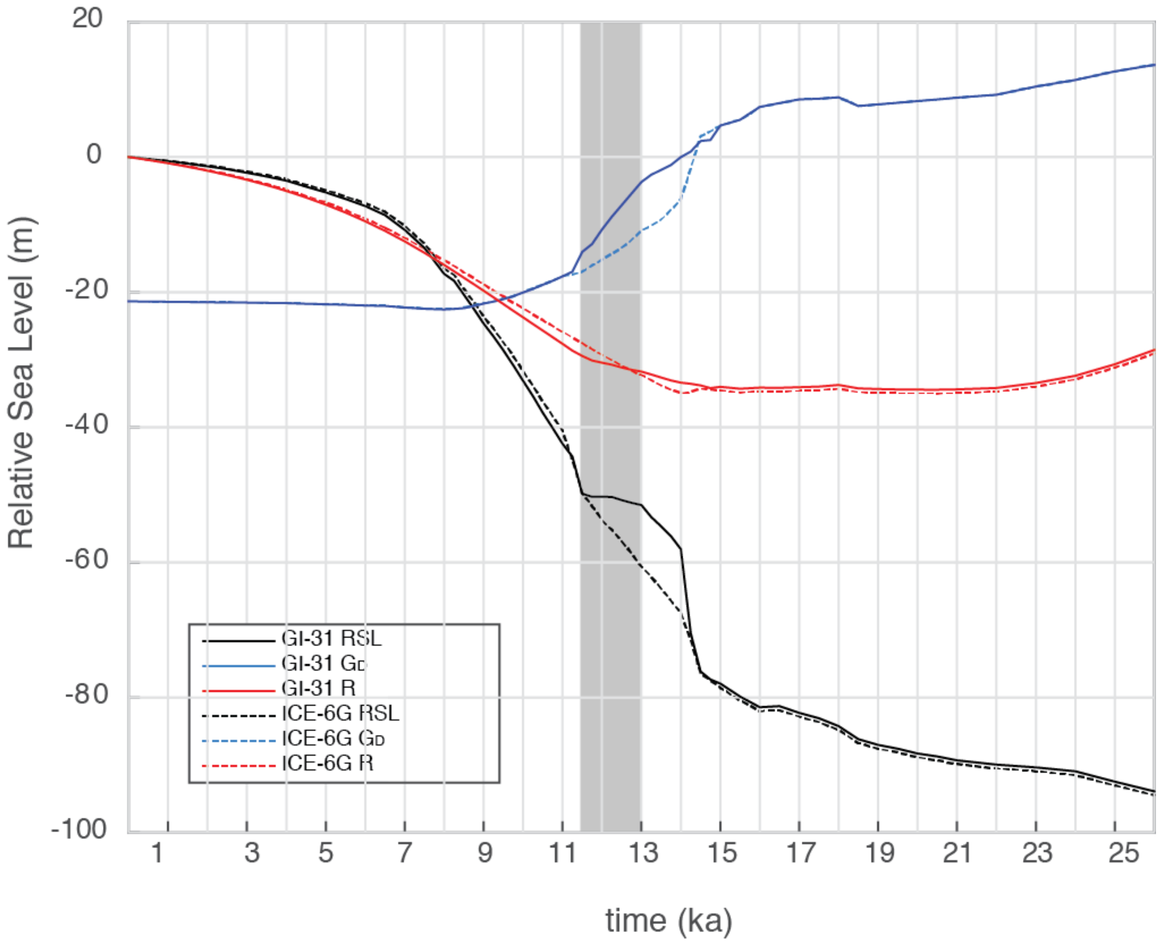

Fig. S4. Decomposition of total relative sea level at the Bering Strait sill (black lines) into components associated with the direct gravitational effect of the surface load (G_D, blue lines) and crustal deformation, including the local gravitational effect of this deformation (R, red lines). Dashed lines are computed by adopting ice model ICE-6G and solid lines adopt GI-31, as in main text. The gray rectangle highlights the 13-11.5 ka interval.

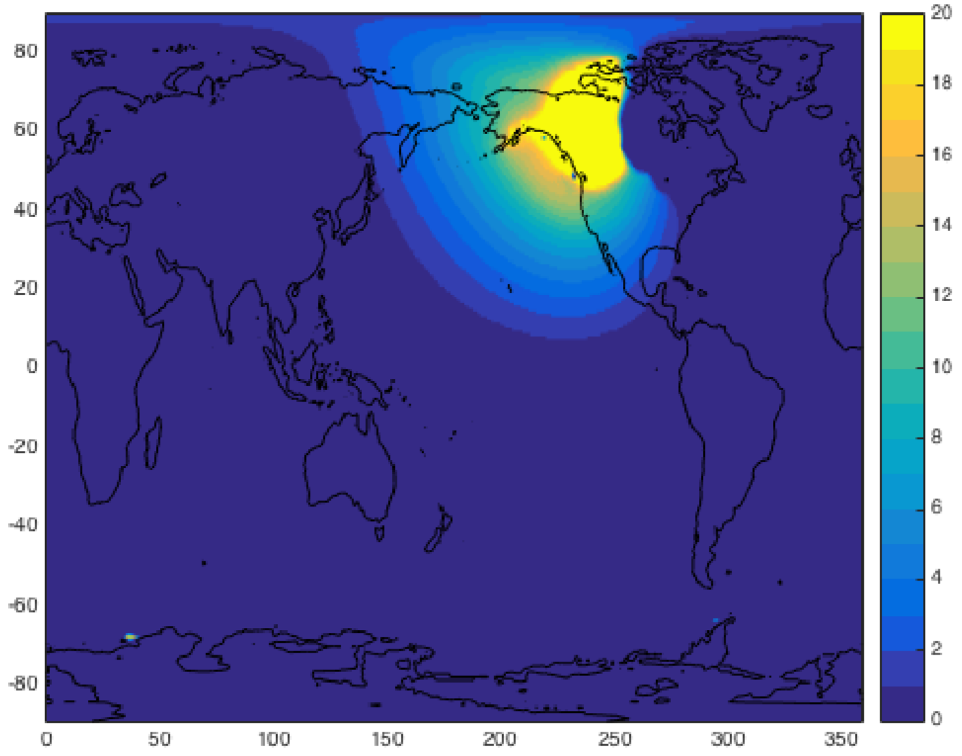

Fig. S5. Map of the difference in relative sea level predictions at 13 ka BP predicted using the GI-31 and ICE-6G ice histories (i.e., GI-31 result minus ICE-6G result) and the Earth model described in the main text.

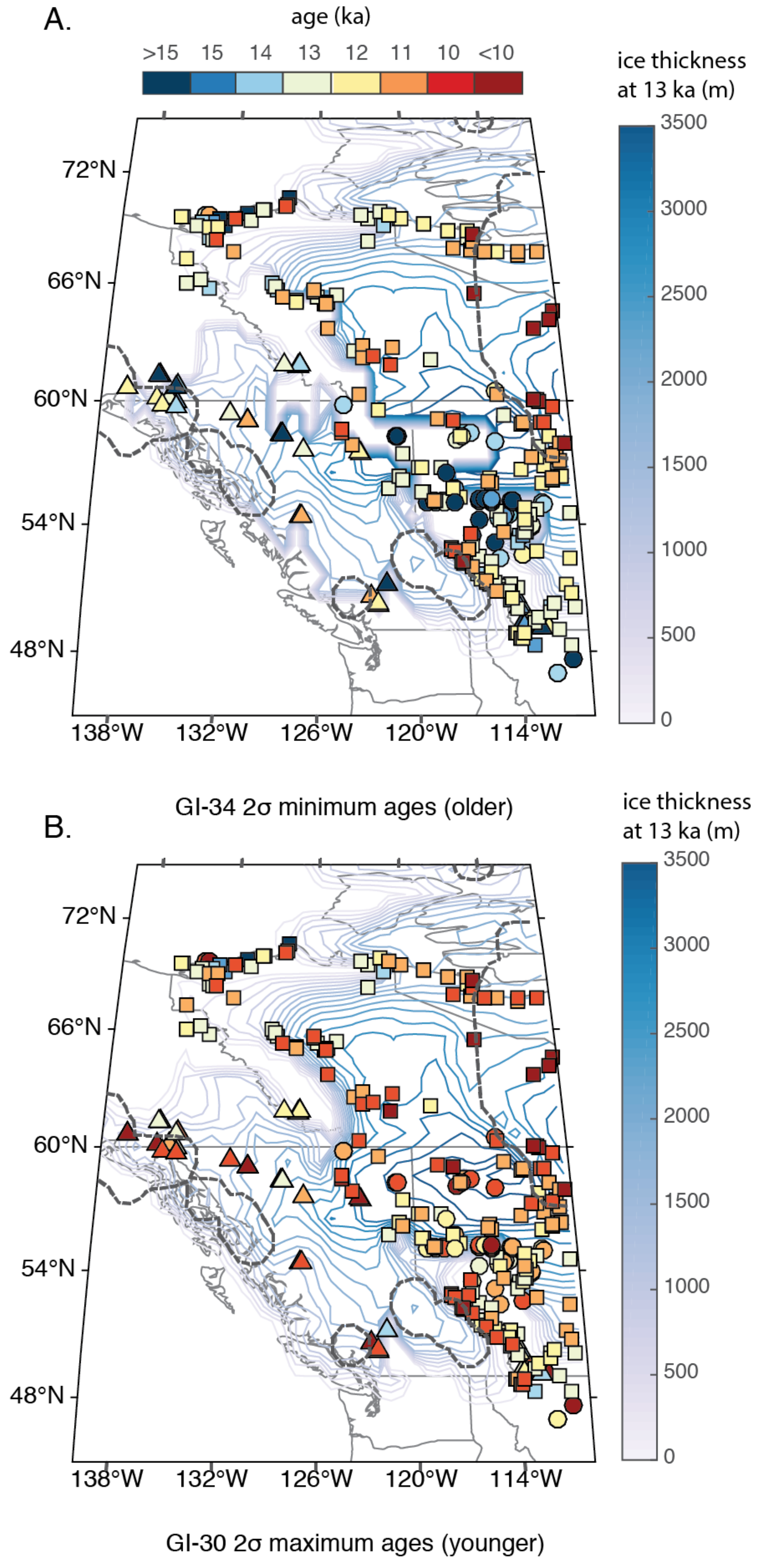

Fig. S6. Ice melting scenario from 13-11.5 ka for GI-34 (**A**) and GI-30 (**B**). Contours represent ice thickness at 13 ka (right-hand colorbar). Squares represent calibrated radiocarbon ages, circles

represent luminescence ages, and triangles represent cosmogenic ages. Interior colors represent ages rounded to nearest integer (top colorbar). Dotted gray lines represent the limits of ice extent at 11.5 ka.

Table S1. Compilation of ice models used in this study.

Model	Melt region				Timing of melt (ka)				ΔRSL	Supp Figure
	CIS/ w. LIS	e. LIS	AIS	FIS	13-12.5	12.5-12	12-11.5	11.5-11		
GI-15	✓				✓	✓	✓		1.2	7A/B black
GI-12	✓				✓✓	✓	✓		-0.7/2.2	7A orange
GI-14	✓				✓	✓	✓	✓	1.5m/ky	7A green
GI-18	✓					✓	✓		0.2m/ky	7A blue
GI-19	✓	✓			✓	✓	✓		2.2	7A purple
GI-22	✓	✓			✓	✓	✓		0.68	7B red
GI-23	✓				✓	✓	✓		4.85	7B blue
GI-24	✓	✓			✓	✓	✓		2.5, 3, 5.1	7B purple, yellow, orange
GI-25	✓				✓	✓	✓		0.93	7B green
GI-26	✓				✓	✓	✓		6.28	7B light blue
GI-27	✓			✓	✓	✓	✓		4.28	7B crimson
GI-28	✓		✓		✓	✓	✓		3.71	7B magenta
GI-30	✓				✓	✓	✓		1.2	13 blue
GI-34	✓				✓	✓	✓		1.7	13 green
GI-31 (main text)	✓				✓	✓	✓		1.3	Main text Fig. 2 black

A.

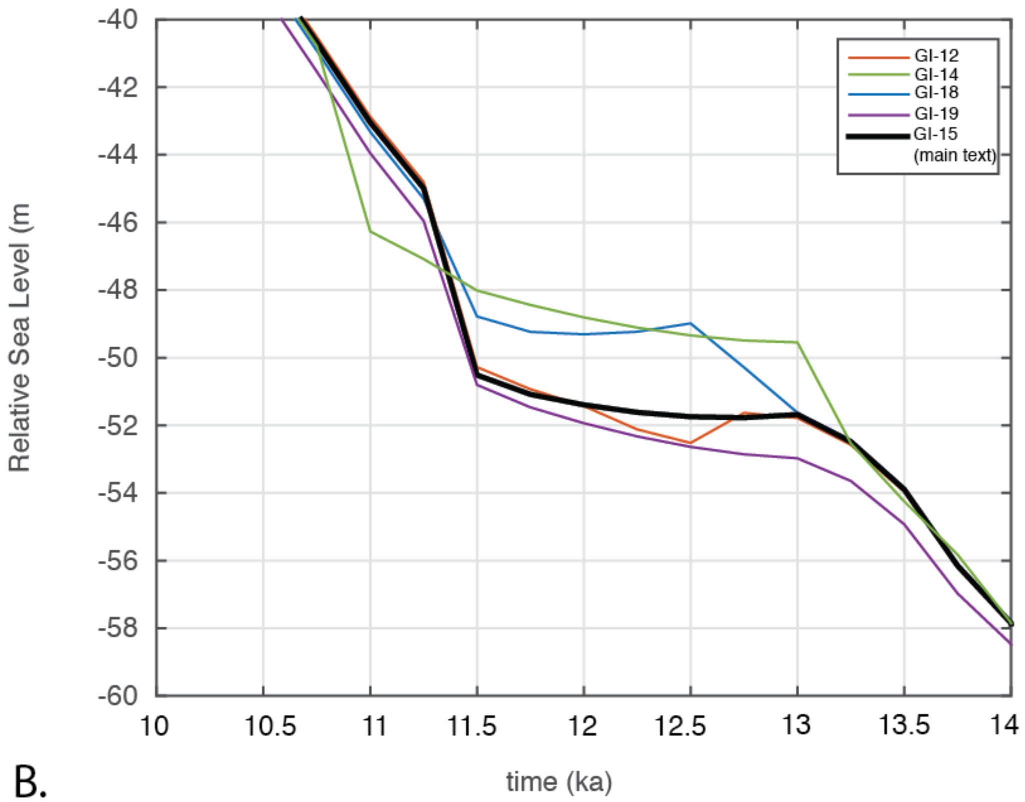

GI-12
GI-14
GI-18
GI-19
GI-15
(main text)
Relative Sea Level (m
time (ka)

B.

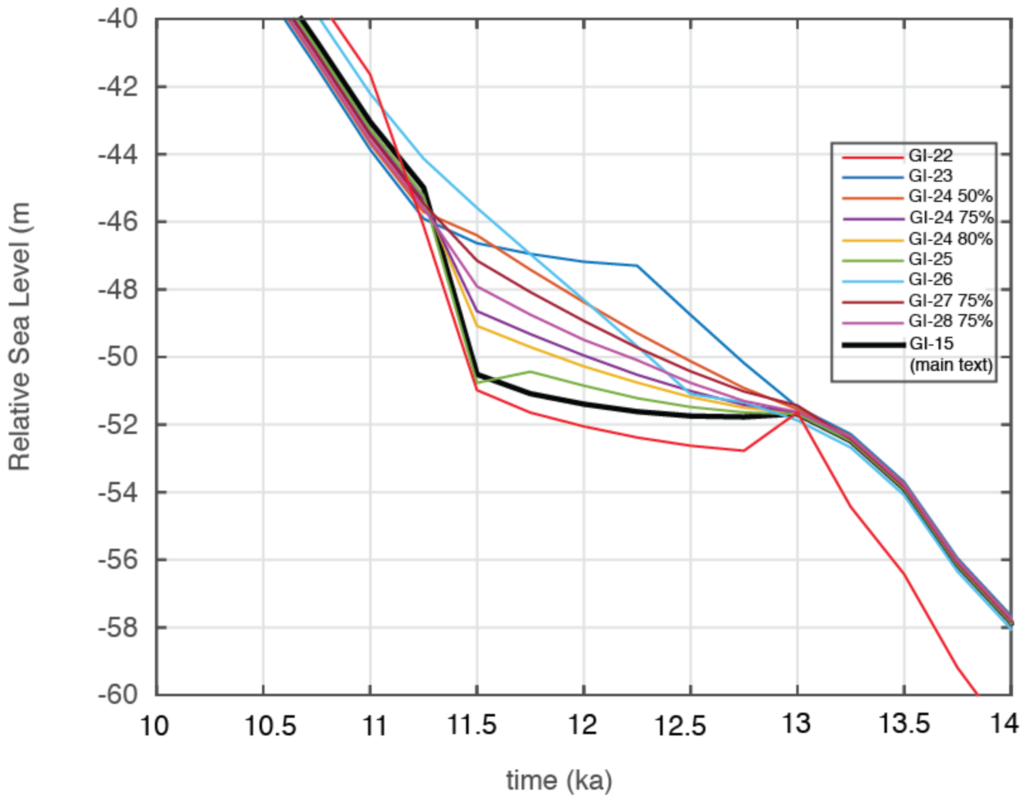

GI-22
GI-23
GI-24 50%
GI-24 75%
GI-24 80%
GI-25
GI-26
GI-27 75%
GI-28 75%
GI-15
(main text)
Relative Sea Level (m
time (ka)

Fig. S7. Relative sea level predictions based on a suite of ice models, testing the sensitivity of the predictions to changes in the regional distribution and duration of ice melt. (See text for a full description of the different ice models.) **A.** Ice models GI-12, GI-14, GI-18, GI-19, GI-15. **B.** Ice models GI-22, GI-23, GI-24 (50%,75%,80%), GI-25, GI-26, GI-27, GI-28, and GI-15).

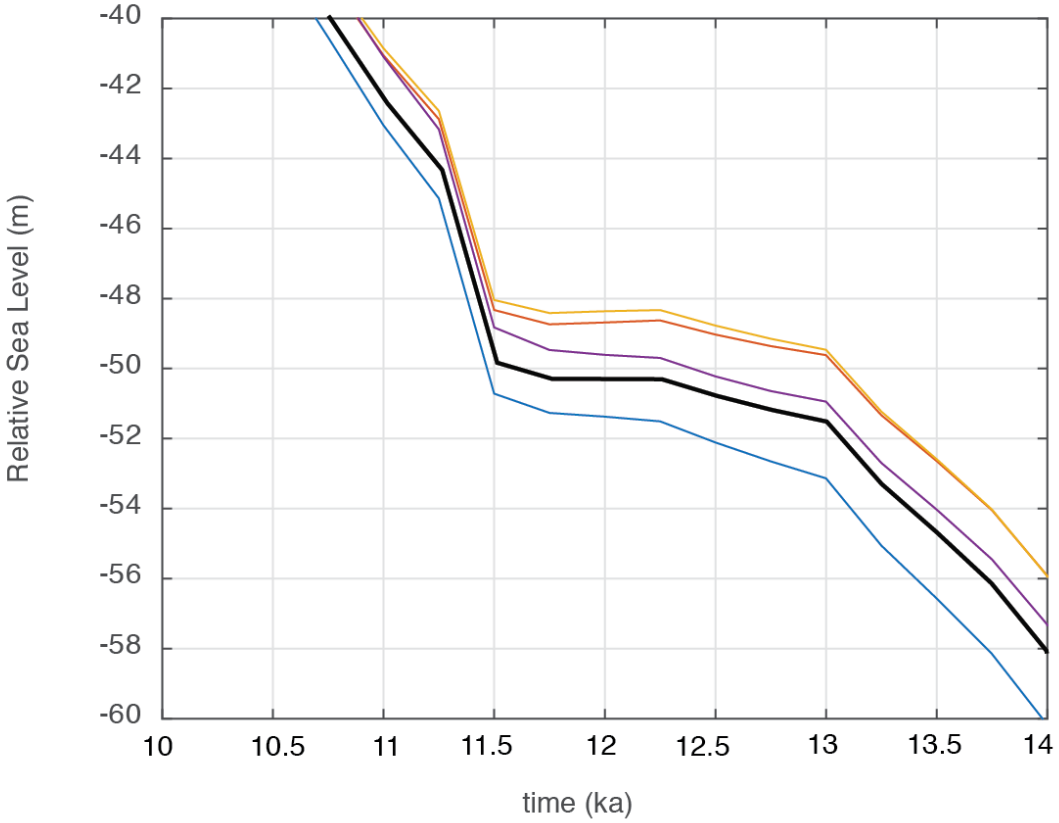

Fig. S8. Earth model sensitivity. Relative sea-level predictions based on ice model GI-31 using a suite of Earth models. Solid black line is the prediction based on the Earth model adopted in main text. Yellow, blue, orange, and purple lines are based on Earth models with a lithospheric thickness, upper mantle viscosity and lower mantle viscosity of: (96 km, 5 $x10^{20}$ Pa s, $5x10^{21}$); (48 km, 5 $x10^{20}$ Pa s, $3x10^{21}$ Pa s); (48 km, 5 $x10^{20}$ Pa s, $7x10^{21}$); (48 km, 3 $x10^{20}$ Pa s, $5x10^{21}$).

Table S2. Calibrated radiocarbon ages using a larger uncertainty on reservoir ages than in main text (ΔR = 300 ± 200 years).

Reference	Site name	Latitude	Longitude	Material	Marker depth (m)	Raw 14C age (yrs)	Raw age uncertainty (1[illegible])	ΔR	ΔR uncertainty (yr)	Calendar Yr	1σ - yr	1σ + yr	Calibration curve
Jakobsson et al., 2017	4PC-1	72.8	175.7	mollusc	124.07	10200	30	300	200	10850	230	260	Marine13
Keigwin et al., 2016	JPC02	67.4	165.6	E. excavatum	53.455	10900	140	300	200	11870	470	390	Marine13
Hill et al., 2008	VBC03*	70.7	165.4	marine bivalve (Portlandia)	60.42	11500	765	300	200	12520	1100	910	Marine13
	JPC10	70.8	165.5	marine bivalve	59.2	10200	55	300	200	10850	230	260	Marine13
Elias et al., 1996	85-69	70	165.7	screened peat	44.95	11000	60	0	0	12870	90	80	Intcal13
Dyke & Savelle, 2001				Bowhead whale bone		10210	70	300	200	10860	240	260	Marine13
Dyke et al., 1996				M. Balthica		11400	100	300	200	12570	280	530	Marine13
England & Furze, 2008				C. kurriana		12,380	110	300	200	13560	240	500	Marine13
England & Furze, 2008				C. kurriana		12,170	25	300	200	13340	210	440	Marine13
England & Furze, 2008				C. kurriana		11,800	70	300	200	12980	220	380	Marine13

* *Portlandia Arctica* raw ^{14}C age includes correction (800±700 yr subtracted from published raw ^{14}C age).

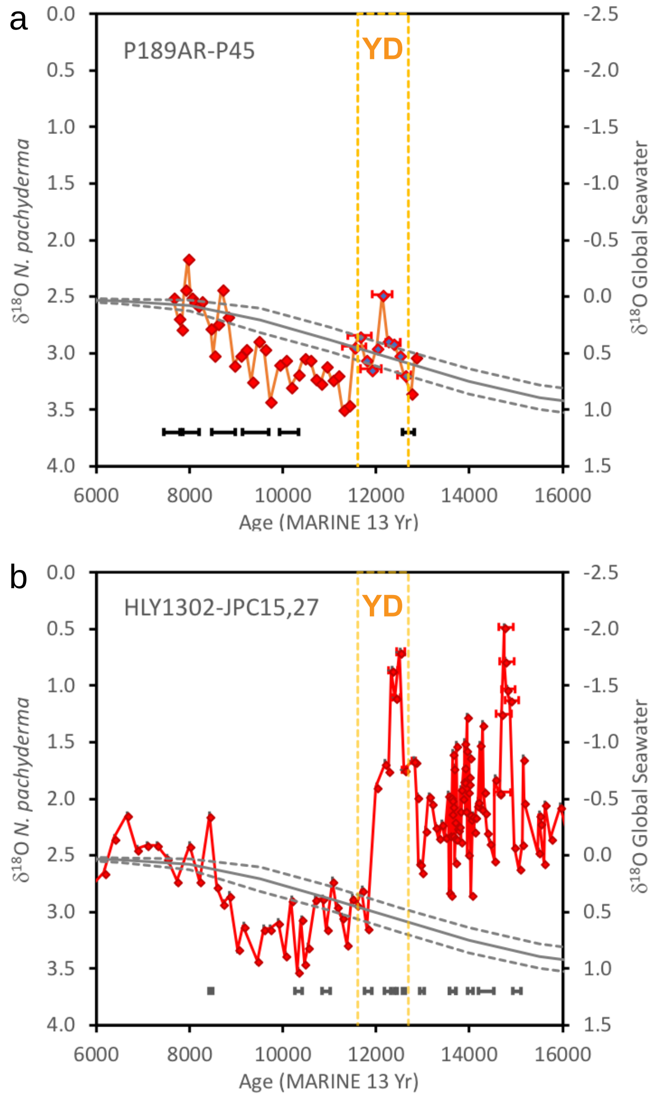

Fig. S9. Oxygen isotope record from planktonic foraminifera left-coiling *N. pachyderma* (red symbols and lines) from the Mackenzie River delta region of the Arctic Ocean in a) sediment core P189AR-P45 [23] and b) sediment core HLY1302-JPC15,29 [24]. For comparison, a reconstruction of the global average ice-volume component of $\delta^{18}O$ (global seawater) is shown as a gray line (uncertainty envelope represented by dashed lines)[47]. Vertical bar demarcates the Younger Dryas (YD) interval. Age models are recalculated here using ^{14}C dates calendar corrected based on our adopted reservoir age constraints (black bars span 2σ uncertainty). Age uncertainties of BCHRON Bayesian age model applied to the $\delta^{18}O$ samples in the intervals of anomalously low $\delta^{18}O$ (i.e., high freshwater runoff) are shown as red horizontal bars. Low values of $\delta^{18}O$ relative to the global trend are consistent with

increased flux of freshwater from melting continental ice. The apparent freshwater anomalies are relatively subdued in P189AR-P45, yet strong in HLY1302-JPC15,29, which also suggests a series of earlier events. The two cores agree that an interval of relatively high $\delta^{18}O$ (implying cessation or re-routing of freshwater runoff to the region) occurred between ~12,000 and ~9000 yr BP.

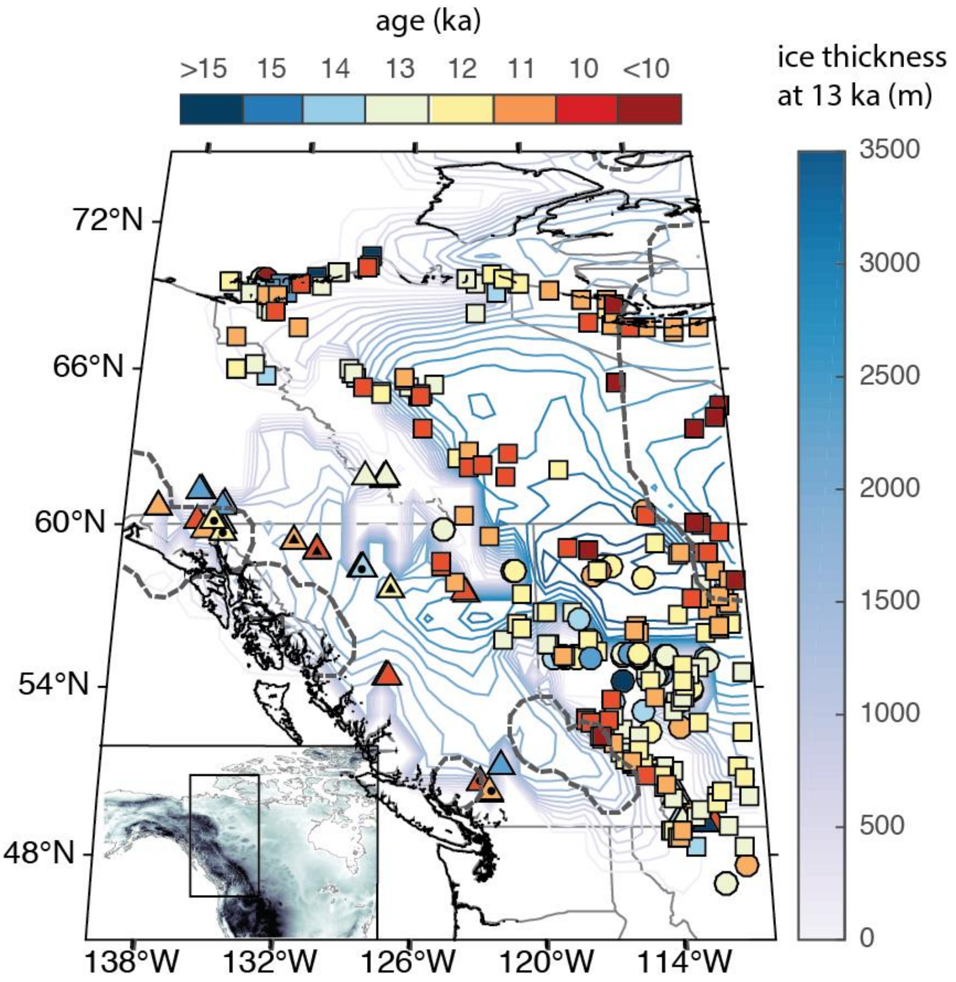

Fig. S10. The location of cirque (dots) and valley (triangle) glacier moraines in the Menounos *et al.* (*49*) study is shown on Fig. 4. See Supplementary Material 7 for a discussion of these ages.

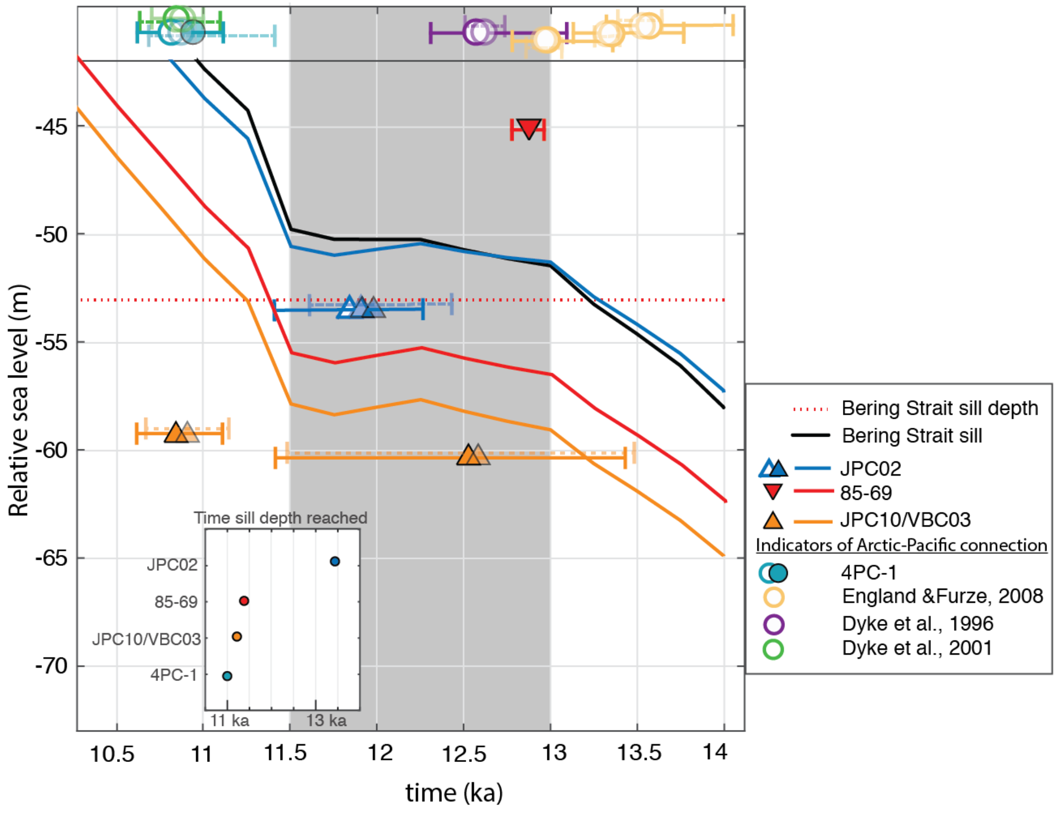

Fig. S11. Relative sea level predictions for sites in the Bering Strait region compared with observations using radiocarbon dates calibrated with additional uncertainty. Translucent markers with dotted uncertainty bars represent ages adopted in main text (as in Fig. 2), solid markers and uncertainty bars are ages calibrated with ΔR = 300±200 yr. Horizontal bars denote 1σ uncertainties.

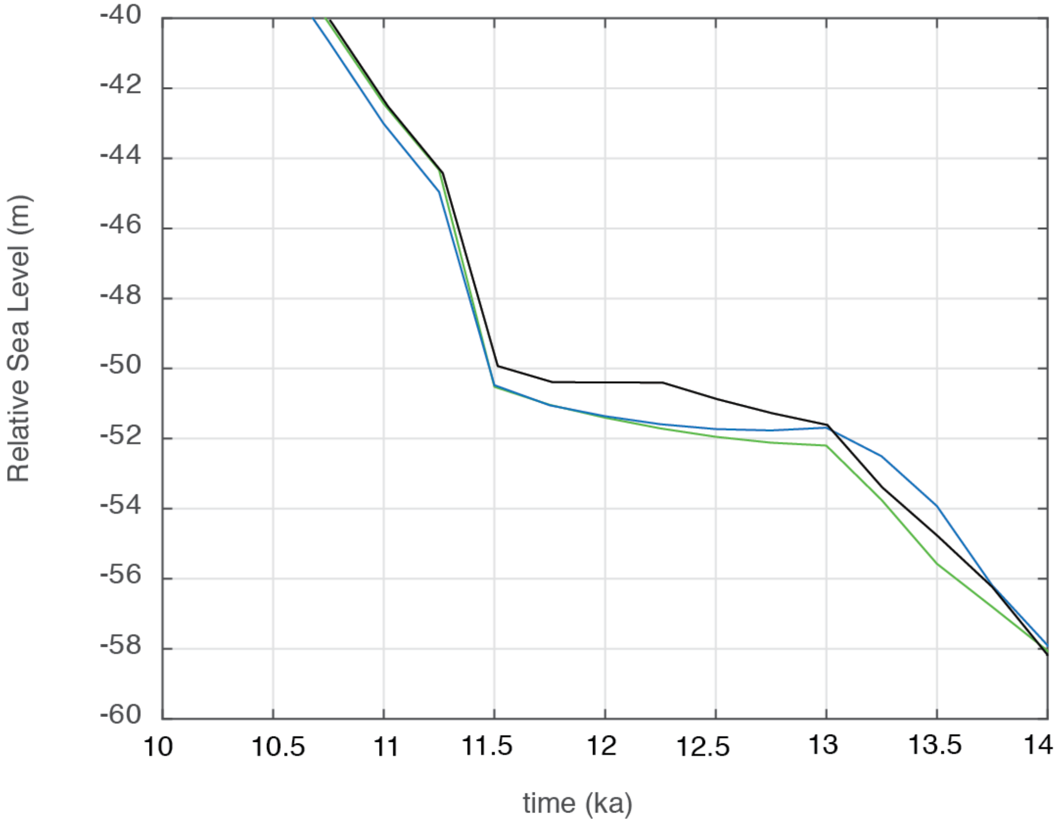

Fig. S12. Relative sea level predictions using ice history GI-31 (as in the main text; black), GI-30 (2σ uncertainty maximum ages; blue), and GI-34 (2σ uncertainty minimum ages; green). See fig. S4 for ice-melting scenarios for GI-30 and GI-34. All simulations adopt the Earth model described in the main text.

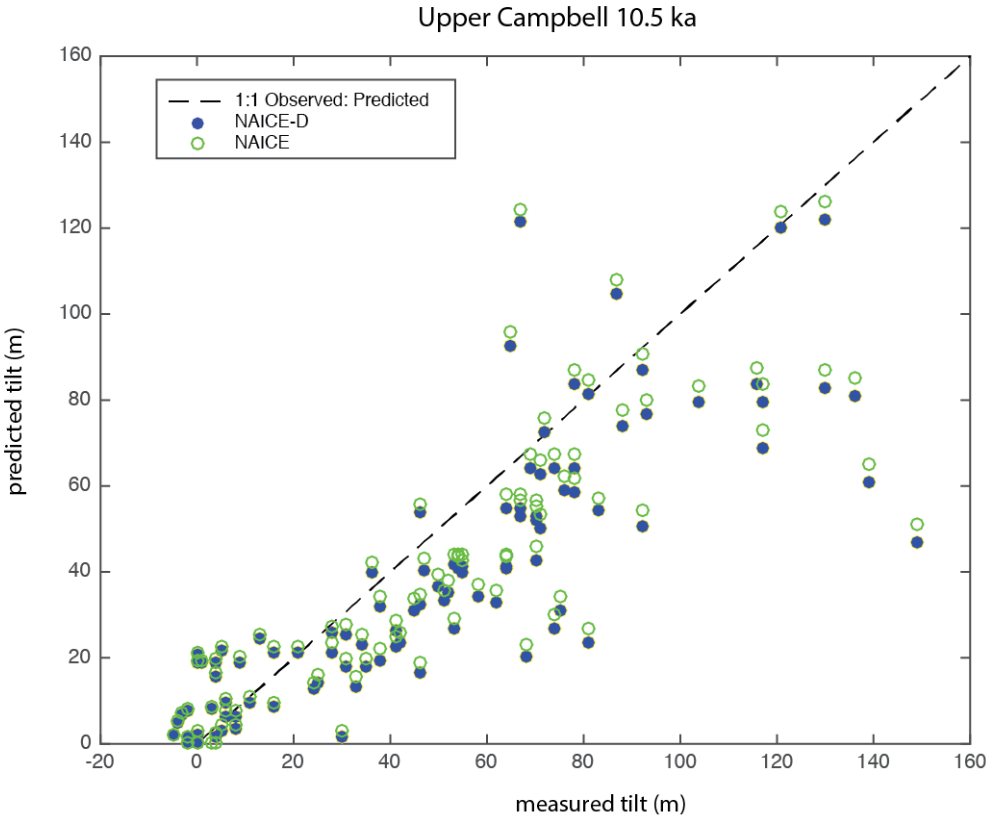

Fig. S13. Comparison of measured and predicted tilt using ice history NAICE-D (blue filled circles) and NAICE (green circles).

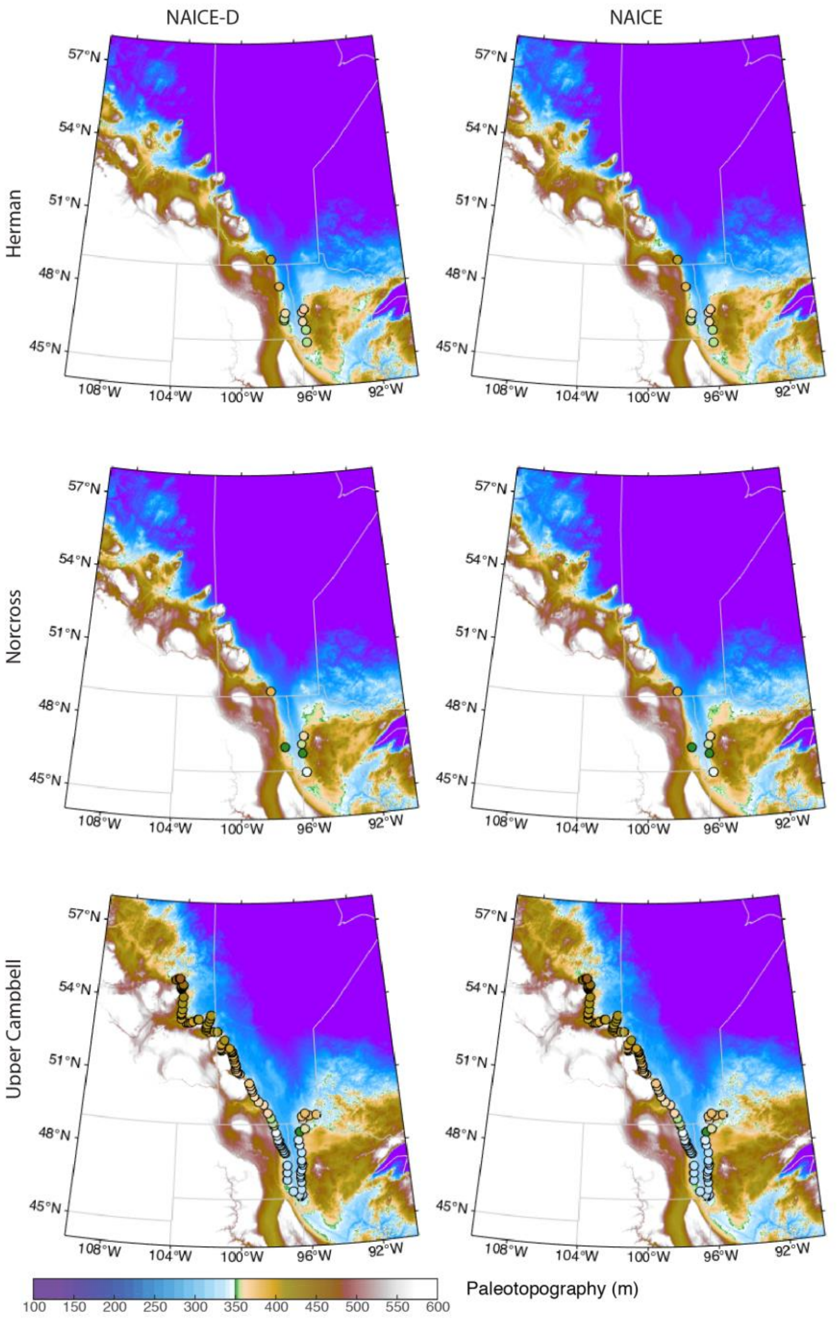

Fig. S14. Paleotopography compared to observed shoreline elevations. Paleotopography is predicted using NAICE-D (left) and NAICE(right) compared with observed elevation of strandline (circles).

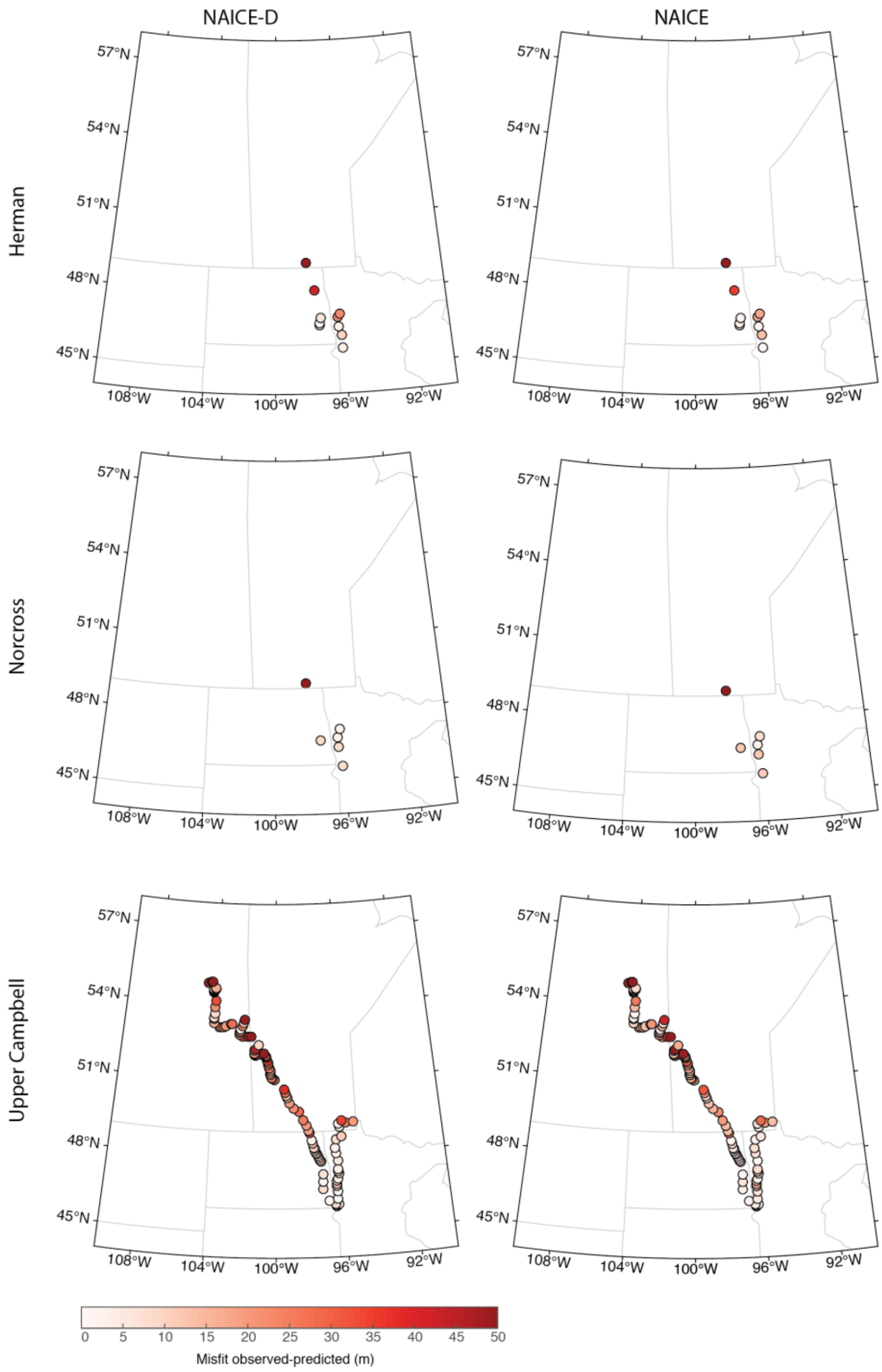

Fig. S15. Misfit between the observed and predicted paleotopography for each glacial Lake Agassiz shoreline for NAICE-D (left) and NAICE (right).

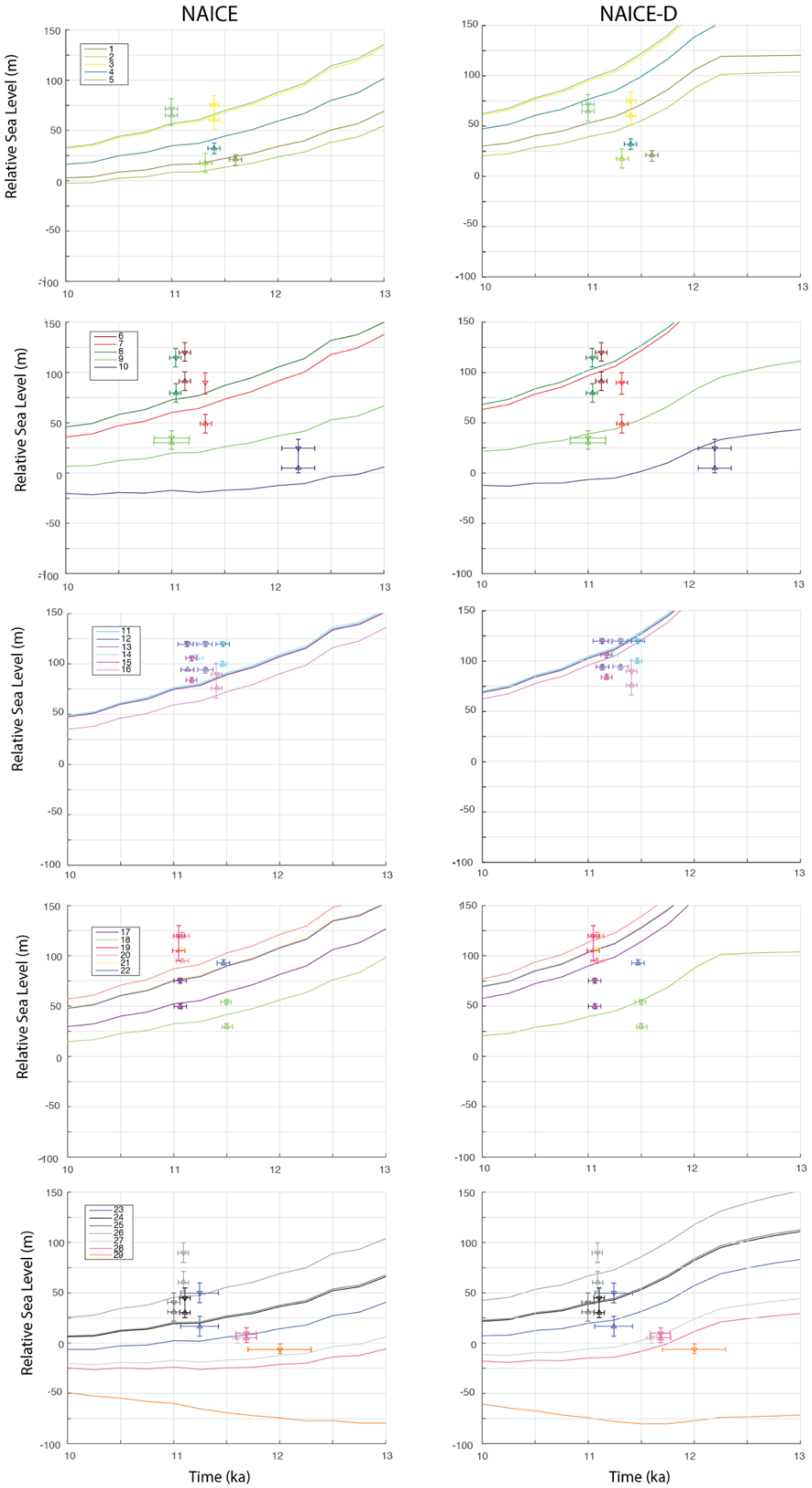
NAICE
NAICE-D
Relative Sea Level (m)
Time (ka)
1
2
3
4
5
6
7
8
9
10
11
12
13
14
15
16
17
18
19
20
21
22
23
24
25
26
27
28
29

Fig. S16. Relative sea level predictions adopting NAICE (left) and NAICE-D (right) compared with relative sea level markers in the Canadian Arctic older than 11 ka (sites labeled 1-29 from Gowan et al.[28]).

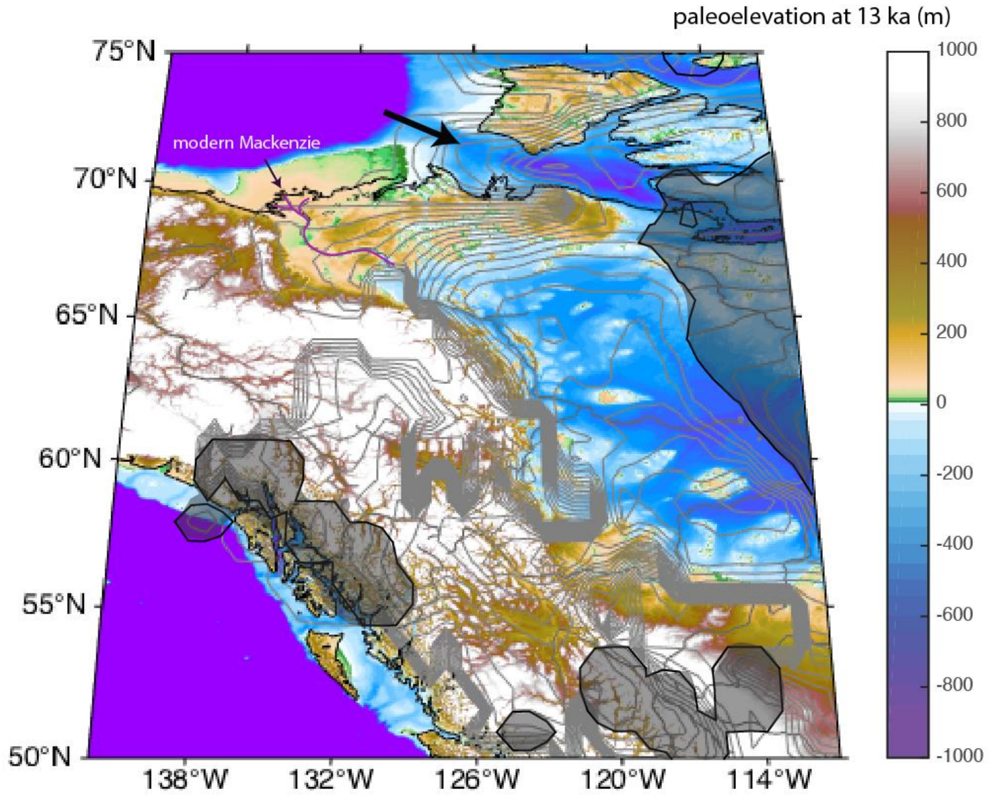

Fig. S17. Possible marine retreat of ice sheet. Modeled paleoelevation at 13 ka using GI-31 ice history. Contours show the margin and thickness of ice at 13 ka. Shaded regions show ice margin at 11.5 ka. Region with reverse bedrock slope (1m/km) is highlighted by black arrow. This region of ice may have been subject to a marine ice sheet instability, where water at the base of the ice sheet induces melting, causing a rapidly retreating grounding line to induce a large mass loss in this region.

Data file S1. Compilation of ages constraining timing of ice retreat in CIS/Western LIS.
